# From a Traditional Medicinal Plant to a Rational Drug: Understanding the Clinically Proven Wound Healing Efficacy of Birch Bark Extract

**DOI:** 10.1371/journal.pone.0086147

**Published:** 2014-01-22

**Authors:** Sandra Ebeling, Katrin Naumann, Simone Pollok, Tina Wardecki, Sabine Vidal-y-Sy, Juliana M. Nascimento, Melanie Boerries, Gudula Schmidt, Johanna M. Brandner, Irmgard Merfort

**Affiliations:** 1 Pharmaceutical Biology and Biotechnology, Albert-Ludwigs-University Freiburg, Freiburg, Germany; 2 Department of Dermatology and Venerology, University Hospital Hamburg-Eppendorf, Hamburg, Germany; 3 Institute for Experimental and Clinical Pharmacology and Toxicology, Albert-Ludwigs-University Freiburg, Freiburg, Germany; 4 Institute of Molecular Medicine and Cell Research, Albert-Ludwigs-University Freiburg, Freiburg, Germany; 5 German Cancer Consortium (DKTK), Heidelberg, Germany; 6 German Cancer Research Center (DKFZ), Heidelberg, Germany; CNRS-University of Toulouse, France

## Abstract

**Background:**

Birch bark has a long lasting history as a traditional medicinal remedy to accelerate wound healing. Recently, the efficacy of birch bark preparations has also been proven clinically. As active principle pentacyclic triterpenes are generally accepted. Here, we report a comprehensive study on the underlying molecular mechanisms of the wound healing properties of a well-defined birch bark preparation named as TE (triterpene extract) as well as the isolated single triterpenes in human primary keratinocytes and porcine *ex-vivo* wound healing models.

**Methodology/Principal Findings:**

We show positive wound healing effects of TE and betulin in scratch assay experiments with primary human keratinocytes and in a porcine *ex-vivo* wound healing model (WHM). Mechanistical studies elucidate that TE and betulin transiently upregulate pro-inflammatory cytokines, chemokines and cyclooxygenase-2 on gene and protein level. For COX-2 and IL-6 this increase of mRNA is due to an mRNA stabilizing effect of TE and betulin, a process in which p38 MAPK and HuR are involved. TE promotes keratinocyte migration, putatively by increasing the formation of actin filopodia, lamellipodia and stress fibers. Detailed analyses show that the TE components betulin, lupeol and erythrodiol exert this effect even in nanomolar concentrations. Targeting the actin cytoskeleton is dependent on the activation of Rho GTPases.

**Conclusion/Significance:**

Our results provide insights to understand the molecular mechanism of the clinically proven wound healing effect of birch bark. TE and betulin address the inflammatory phase of wound healing by transient up-regulation of several pro-inflammatory mediators. Further, they enhance migration of keratinocytes, which is essential in the second phase of wound healing. Our results, together with the clinically proven efficacy, identify birch bark as the first medical plant with a high potential to improve wound healing, a field which urgently needs effective remedies.

## Introduction

The skin primarily functions as a protective barrier against the environment. Therefore, loss of its integrity immediately results in starting complex processes to restore the epidermal barrier function. The repair of wounds is an extremely complex biological process basically divided into three overlapping phases: inflammation, new tissue formation, and remodeling [1]–[4]. The first stage is characterized by hemostasis and by initiating a controlled inflammatory response. Release of various pro-inflammatory cytokines including chemokines and growth factors orchestrates the attraction of macrophages and granulocytes to the wound area to remove dead tissue and to prevent bacterial infection [5]–[8]. The second stage includes migration and proliferation of keratinocytes ( =  reepithelialization) and fibroblasts ( =  formation of granulation tissue and extracellular matrix) as well as angiogenesis. During the third stage old collagen is replaced, apoptosis takes place and a scar is formed. Hence wound healing requires the integration of many complex cellular and molecular events which can be targeted at many points, leading either to accelerated or delayed healing, the latter can lead to chronic wounds [4], [9], [10].

Besides of the conventional remedies, phytomedicines turned out to be an interesting alternative or addendum to beneficially influence the different stages of wound healing. In this context, extracts from birch bark (*Betula alba,* syn. *B. pendula*, Betulaceae family) have gained more and more interest. Birch bark has a long lasting history as a traditional medicinal remedy known already by the North American Indians who wrapped their wounds with birch bark to accelerate wound healing [11]. Recently, efficacy of birch bark preparations has also been proven clinically. A case report described the successful treatment of necrotizing herpes zoster when using a birch bark emulsion [12]. Moreover, birch bark extract was found to be effective in the treatment of two patients suffering from a second degree burning [13]. Notably, an open, blind-evaluated, controlled, prospective, randomized phase II clinical trial including 24 patients revealed that a birch bark preparation significantly accelerated reepithelialization in split thickness skin graft donor sites [14]. All these studies were conducted with a preparation containing an n-heptane dry extract from the outer bark of birch (TE: triterpene extract) [15]. About 97% of the extract is composed of pentacyclic triterpenes with about 87% of betulin as main compound accompanied by minor amounts of lupeol, betulinic acid, oleanolic acid and erythrodiol (for structures see Fig. S1B). Hence these pentacyclic triterpenes are considered as the mainly effective constituents of birch bark. The individual triterpenes themselves have not yet been evaluated for their wound healing efficacy.

Given the positive effect of TE in *in-vivo* wound healing we wanted to elucidate the underlying molecular mechanisms of its wound healing properties as well as of the isolated single triterpenes (betulin, lupeol, betulinic acid, oleanolic acid and erythrodiol). We demonstrate that TE and betulin influence the inflammatory phase of wound healing by upregulating pro-inflammatory cytokines, chemokines and cyclooxygenase-2 (COX-2) in human primary keratinocytes. Exemplarily, we confirm upregulation in the *ex-vivo* pig wound healing model for IL-6 and COX-2. We provide evidence for COX-2 and IL-6 that their mRNA increase is due to an mRNA stabilizing effect, a process in which p38 MAPK and HuR (human antigen R) are essentially involved. We demonstrate that TE, betulin, lupeol and erythrodiol increase the formation of actin filopodia, lamellipodia and stress fibers, processes that are dependent on the activation of Rho GTPases. Finally, we show in the porcine *ex-vivo* model that TE improves epidermal regeneration and accelerates the repair of the epidermal barrier function.

## Results

### TE and betulin exhibits wound healing effects in a porcine *ex-vivo* wound healing model (WHM)

**Figure 1:**
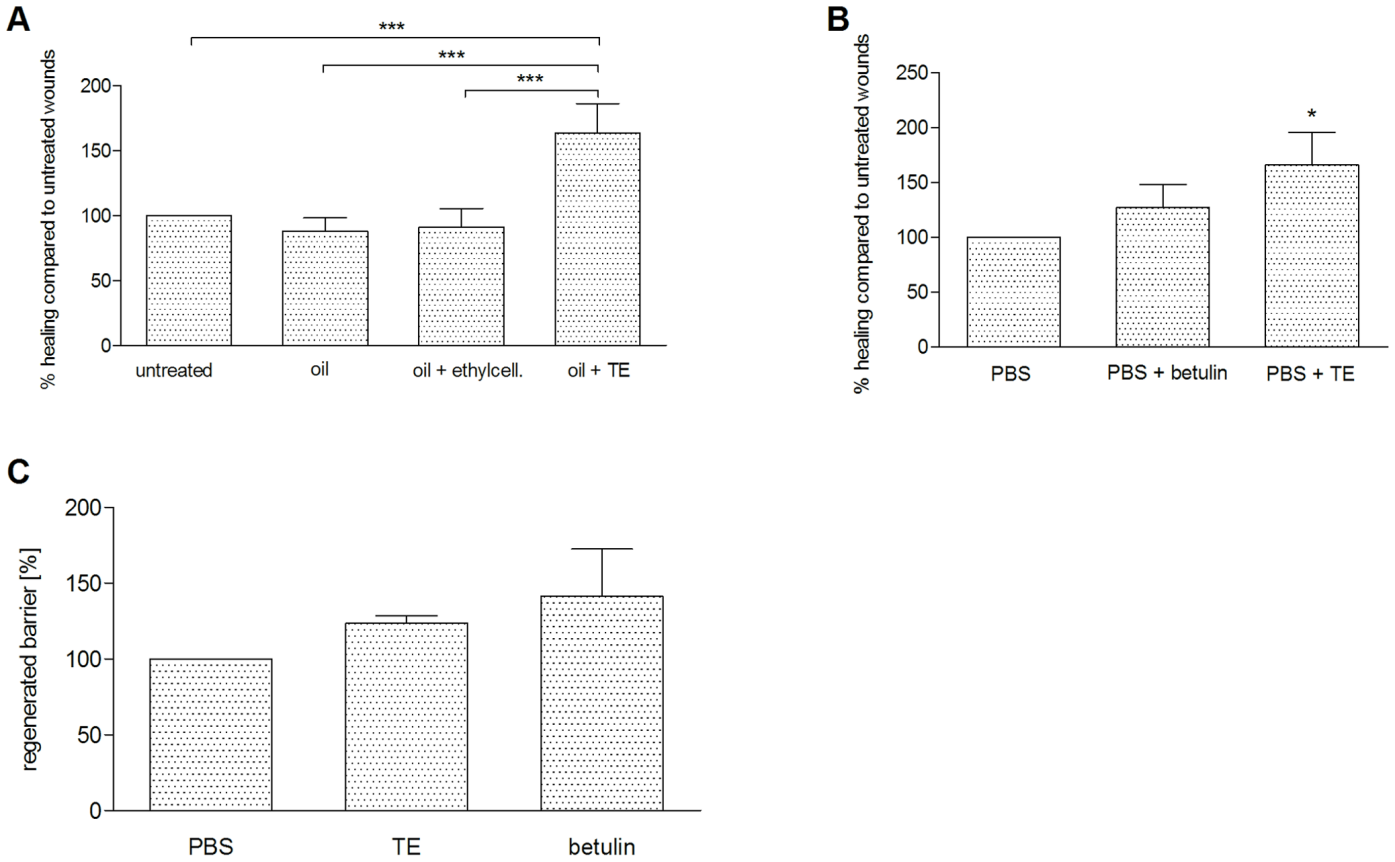
Wound healing progress in WHM. Effect of TE and betulin on reepithelialization in the porcine *ex-vivo* WHM. (A) Reepithelialization in WHM treated with 10% TE in sunflower oil (oleogel) compared to sunflower oil alone, sunflower oil with ethylcellulose and untreated control 48 h after wounding. (B) Reepithelialization in WHM treated with 10 µg/ml TE and betulin in the concentration as it occurs in 10 µg/ml TE in PBS compared to PBS 48 h after wounding. Reepithelialization of the various models was normalized to untreated control (A) or PBS (B), respectively. Mean ± SEM. (C) Effect of TE and betulin in PBS on barrier regeneration in WHM normalized to barrier regeneration in WHM treated with PBS alone. WHM were treated 72 h after wounding for 24 h. Mean ± SEM. A: n = 7; B: n = 5–7; C: n = 4–5. *p<0.05 and ***p<0.001.

The efficacy of a birch bark preparation (TE-oleogel) has already been proven in humans [14], but the influence of the vehicle control or the main constituent, the triterpene betulin, was not studied separately. Therefore, we systematically investigated the wound healing progress in a porcine *ex-vivo* wound healing model (WHM) using an oleogel with the same composition as used in [14] (10% TE, 90% sunflower oil) compared to sunflower oil alone and sunflower oil in ethylcellulose to mimic the higher viscosity of oleogel compared to oil. We found a significant acceleration of reepithelialization with TE-oleogel compared to the controls after 48 h (Fig. 1A). There was also a significant improvement compared to vaseline (data not shown). Additionally, we analyzed whether the use of TE dissolved in PBS also resulted in a beneficial effect on wound healing in the WHM. This approach allowed us to have a rational basis for our further experiments on the elucidation of the underlying molecular mechanisms of the wound healing properties of TE, because these studies were performed with primary human keratinocytes in culture where the oleogels or the oils can not be used. Indeed, we could show significantly accelerated wound healing with 10 μg/ml TE in PBS compared to PBS alone 48 h after wounding (Fig. 1B). We also observed a beneficial effect with betulin (8.69 μg/mL), which was studied in a concentration as it occurs in 10 μg/ml TE, but the effect was less than that one of TE and statistically not significant. Therefore, it can be assumed that betulin, the main constituent of TE, is not exclusively responsible for the effect observed (Fig. 1B).

### TE promotes formation of the skin barrier

A major goal of wound healing is the restoration of the skin barrier to protect the body from the invasion of pathogens. Therefore, we investigated whether 10 μg/ml TE and 8.69 μg/mL betulin, respectively, improve skin barrier function by using a dye penetration assay in our *ex-vivo* WHM (Fig. 1C). Application of TE and betulin 72 h after wounding for 24 h resulted in an improved skin barrier function. This was the same when TE was used directly after wounding for 4 days (data not shown).

### TE and betulin increase mRNA of proinflammatory mediators in primary human keratinocytes

**Figure 2:**
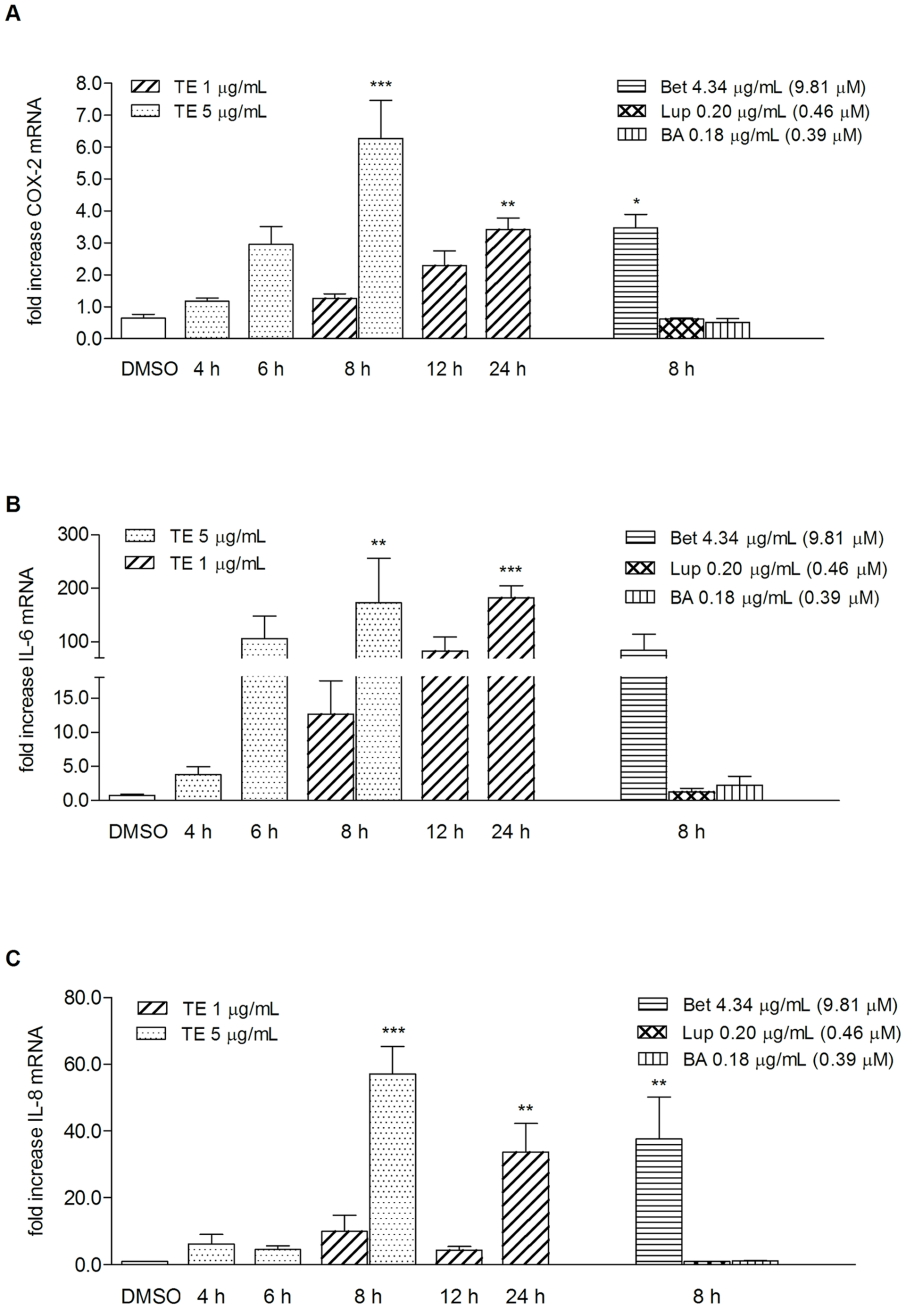
Birch bark (TE) and betulin differently influence mRNA of COX-2, IL-6 and IL-8 in human primary keratinocytes. COX-2 (A), IL-6 (B) and IL-8 (C) mRNA are upregulated in response to birch bark (TE) and betulin (Bet), but not to lupeol (Lup) and betulinic acid (BA). Time course of mRNA expression in response to TE (1 and 5 µg/mL) measured by qRT-PCR. The isolated triterpenes (Bet, Lup, BA) were measured in that concentration in which they occur in 5 µg/mL TE extract. Values represent means of at least three independent experiments ± SEM. *p<0.05, **p<0.01, ***p<0.001 versus control (DMSO).

Disruption of the epidermal barrier induces the release of various pro-inflammatory mediators, such as cytokines, enzymes or growth factors from keratinocytes and platelets [2], [5], [6]. Both events promote the recruitment of granulocytes and macrophages to the site of injury. These cells augment the inflammatory phase by induction of further proinflammatory mediators [2], [16]. In this context, COX-2, IL-6 and IL-8 have been shown to be upregulated and to play crucial roles in reepithelialization and angiogenesis [17]–[20]. To evaluate whether this initiating phase can be accelerated by increasing the amount of these pro-inflammatory mediators, primary human keratinocytes were treated with two different concentrations of TE (1 and 5 μg/mL) and its main triterpenes, betulin, lupeol, and betulinic acid, respectively, and their impact on mRNA expression at different time points was evaluated. The triterpenes were studied in concentrations in which they occur in 5 μg/mL of the studied extract. mRNA of COX-2 significantly increased after an 8 h treatment with 5 μg/mL TE (6.3-fold ±1.2) and 4.34 μg/mL ( = 9.81 μM) betulin (Bet, 3.5-fold ±0.4) (Fig. 2A). Treatment with 1 μg/mL of TE resulted in lower levels of COX-2 and were significant after 24 h (3.4-fold ±0.3). The effect on IL-6 mRNA expression was much more pronounced with a fold increase of 172.4±83.3 for TE (5 μg/mL) and of 84.3±29.9 for betulin (4.34 μg/mL) after 8 h (Fig. 2B). Again, the effect was dose-dependent, i.e. TE in the concentration of 1 μg/mL caused lower effects (12.7-fold ±4.9 after 8 h), which were time dependently upregulated (82.1-fold ±26.8 for 12 h, 181.9-fold ±22.9 for 24 h) and significant after 24 h. Studies on IL-8 mRNA expression resulted in a 57.1±8.3 fold increase at a concentration of 5 μg/mL TE and of 37.6±12.6 at 4.34 μg/mL of betulin after 8 h. The lower concentration of 1 μg/mL TE gave less high levels of IL-8 mRNA with significance at 24 h (fold increase of 33.7±8.6) (Fig. 2). The triterpenes lupeol and betulinic acid exhibited no effects on the mRNA of these three mediators.

### TE and betulin differently influence mRNA of further mediators in human primary keratinocytes

Subsequently, the influence of TE on further molecules involved in the wound healing process was studied. Whereas mRNA levels of IL-1β remained unchanged when primary keratinocytes were treated with 1 μg/mL TE for 4, 6, 8, 12 and 24 h (Fig. S2A), levels of TNF-α mRNA increased significantly at 12 h and decreased again at 24 h (Fig. S2B). As mentioned above, these cytokines are important for initiating the wound healing process. Transforming growth factor-β (TGF-β) was significantly affected by TE resulting in an increase of about 2.6-fold ±0.4 at 8 h (Fig. S2C). This mediator regulates the recruitment of inflammatory cells and macrophages and the formation of granulation tissue by increasing the expression of genes associated with extracellular matrix formation [2], [3]. The effect of TE and betulin on mRNA of the transcription factor NF-E2-related factor 2 (Nrf2), the antimicrobial peptide human beta-defensin 3 (hBD3) as well as the matrix metalloproteinases (MMPs) MMP-2 and MMP-9 were not significant (Fig. S2).

### TE increases mRNA levels of IL-6 and COX-2 in the WHM

**Figure 3:**
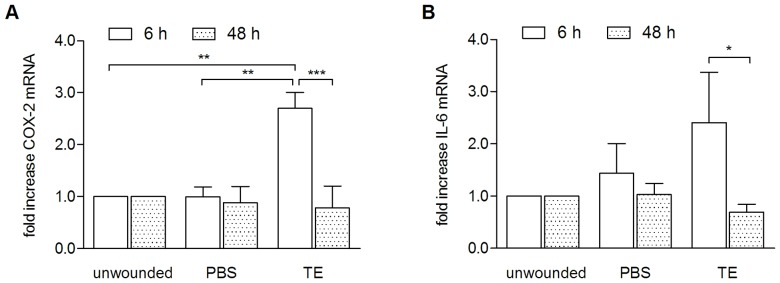
TE upregulates mRNA of IL-6 and COX-2 in an *ex-vivo* wound healing model 6 h after wounding and treatment with 10 µg/ml TE, after 48 h the levels decreased to normal levels. Values represent means of at least three independent experiments ± SEM. *p<0.05, **p<0.01, ***p<0.001 as indicated.

To control whether upregulation of pro-inflammatory mediators found in primary keratinocytes can also be demonstrated in a 3D wound, we exemplarily determined the effect of TE on mRNA level of IL-6 and COX-2 in the porcine *ex-vivo* wound healing model. After treatment with 10 µg/mL TE for 6 and 48 h, mRNA was isolated and quantified by qRT-PCR. A 10 time higher concentration was used to consider the different condition concerning bioavailability in the *ex-vivo* model and also to be in line with the wound healing experiments (Fig 1B). An increase of IL-6 and COX-2 mRNA 6 h after wounding, which significantly decreased after 48 h was observed (Fig. 3).

### TE and betulin increase protein levels of various pro-inflammatory mediators

**Figure 4:**
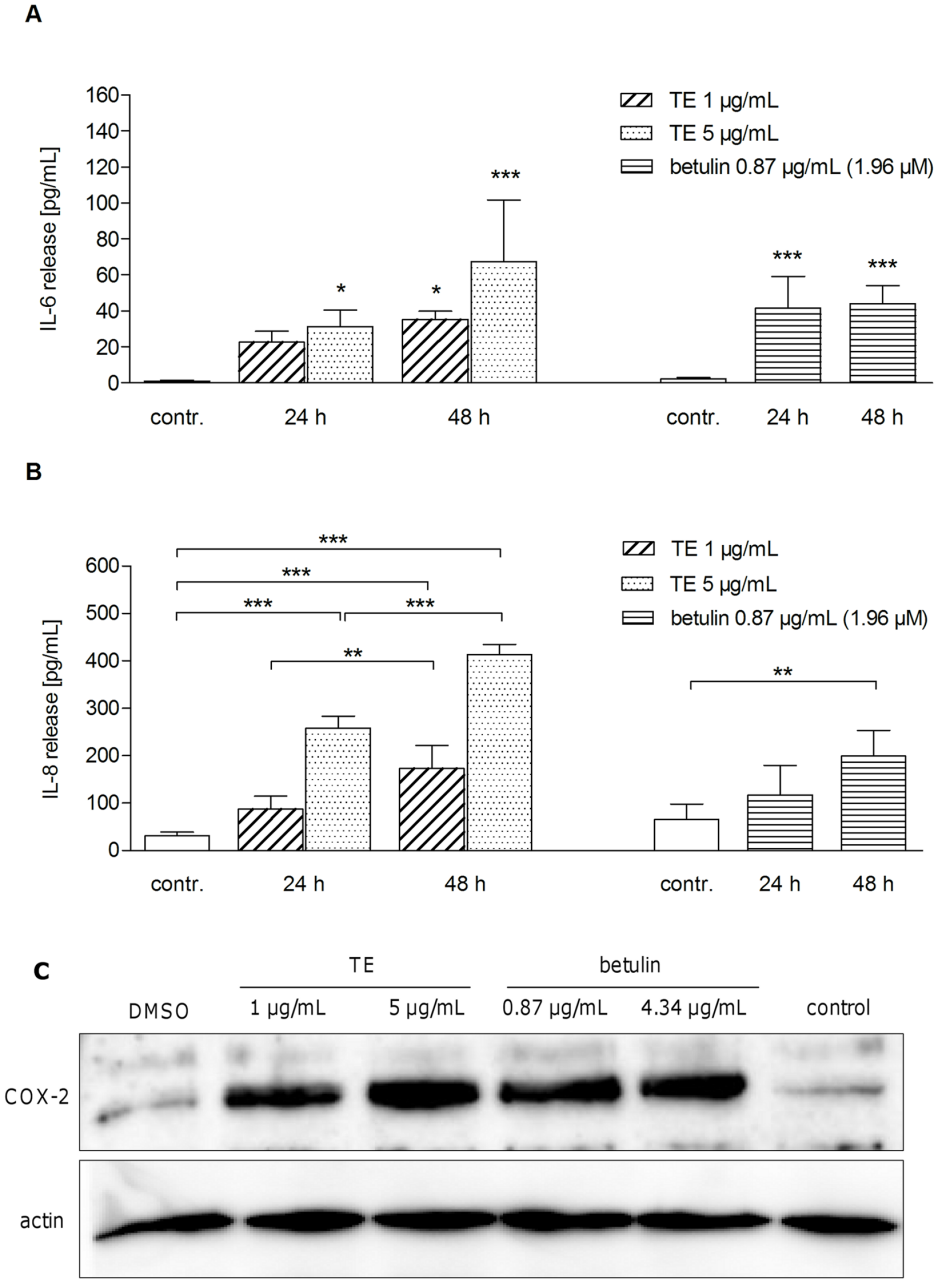
TE and betulin (Bet) enhance release of IL-6 (A) and IL-8 (B), as well as formation of COX-2 protein (C) in primary human keratinocytes. Measurement of IL-6 (A) and IL-8 (B) release in response to TE (1 and 5 µg/mL) and betulin (0.87 µg/mL, which is in 1 µg/mL TE) measured by ELISA. Values represent means of at least three independent experiments ± s.d. (*p<0.05, **p<0.01 and ***p<0.001 versus control). (C) COX-2 protein was measured by Western blot analysis after 24 h treatment with TE (1 and 5 µg/mL) and the respective betulin concentration (0.87 and 4.34 µg/mL). DMSO (0.1%, v/v) served as a solvent control. A representative Western blot is shown, n = 3.

To confirm enhanced levels of pro-inflammatory mediators induced by TE and betulin also on the protein level, firstly the effect on IL-6 and IL-8 release was studied in human primary keratinocytes by ELISA. Supernatants were collected after 24 h and 48 h of incubation with either TE (1 and 5 μg/mL) or betulin (0.87 μg/mL). In both cases enhanced levels of IL-6 and IL-8 were measured compared to control. For IL-6 release highest levels were obtained with 5 μg/mL TE after 48 h (67±34 pg/mL) and with 0.87 μg/mL betulin (51±27 pg/mL) after 24 h (Fig. 4A). IL-8 release was concentration dependent with TE (maximum level of 350±128 pg/mL with 5 μg/mL and 48 h). Lupeol did not influence IL-6 or IL-8 level (data not shown). Protein levels of COX-2 after TE and betulin treatment were determined by Western blot analysis (Fig. 4C). TE and betulin led to an increase in COX-2 protein after 24 h for both tested concentrations (1 and 5 μg/mL TE and 0.87 and 4.34 μg/mL betulin, respectively).

**Figure 5:**
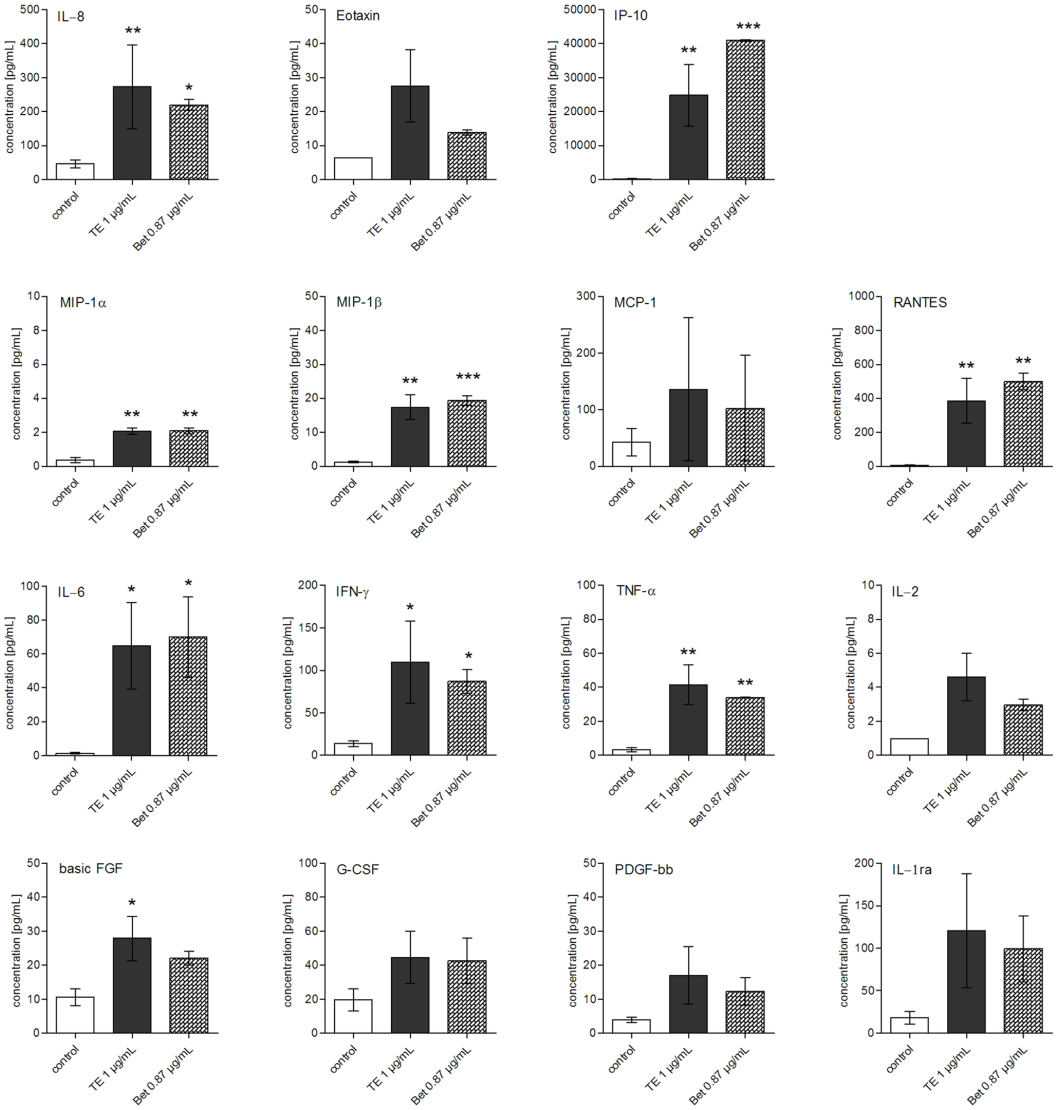
TE and betulin enhance the production of various cytokines, chemokines and growth factors in human keratinocytes after 24 h of incubation. Cells were treated either with TE (1 µg/mL) or betulin (0.87 µg/mL, which is in 1 µg/mL TE) for 24 h. Protein levels of the indicated mediators were determined in the supernatant with the Bio-Plex® Cytokine Assay. Values represent means of two independent experiments ± s.d. *p<0.05, **p<0.01 and ***p<0.001 versus control.

To confirm the data mentioned above with a different method and to gain a more comprehensive overview on further mediators being important in the inflammatory wound healing phase, a Bio-Plex® Cytokine assay was performed. This assay allows the simultaneous measurement of released pro-inflammatory cytokines and growth factors. Only one concentration of TE (1 μg/mL), betulin (0.87 μg/mL  = 1.96 μM) and lupeol (0.04 μg/mL  = 0.09 μM) was tested at 24 h, conditions proven to be suitable from the previous experiments. Altogether, the effect on 27 different mediators including pro-inflammatory [IL-1β, IL-6, interferon-γ (IFN-γ), tumor necrosis factor-α (TNF-α)] and anti-inflammatory [interleukin-1 receptor antagonist (IL-1ra), IL-4, IL-10, IL-13] cytokines, cytokines with various functions (IL-2, IL-5, IL-7, IL-9, IL-12, IL-15, IL-17), chemokines [IL-8, eotaxin, IFN-γ-induced protein-10 (IP-10), monocyte chemotactic protein-1 (MCP-1), macrophage inflammatory protein-1α (MIP-1α), MIP-1β, regulated upon activation, normal T-cell expressed, and secreted (RANTES)] and growth factors [basic fibroblast growth factor (basic FGF), granulocyte colony-stimulating factor (G-CSF), granulocyte macrophage colony-stimulating factor (GM-CSF), platelet derived growth factor-bb (PDGF-bb), vascular endothelial growth factor (VEGF)] was evaluated (Fig. 5 and S3). We could confirm the increase of IL-6 and IL-8 already described above. Significant upregulating effects induced by TE and betulin were also observed for INF-γ, TNF-α, IP-10, MIP-1α and β, RANTES, and basic FGF with IP-10 being released to the highest amount (Fig. 5). The effect on the production of other cytokines and growth factors was not significant (Fig. 5) or negligible (Fig. S3). Lupeol, which was studied at a concentration of 0.04 μg/mL (as it occurs in 1 μg/mL TE), had no effect on the production of these mediators (data not shown).

### TE and betulin have no influence on NF-κB DNA binding, but on mRNA stability

The transcription factor nuclear factor κB (NF-κB) was shown to be involved in the transcriptional regulation of many inflammatory cytokines and chemokines [21]. To elucidate the putative mechanism for elevated amounts of mediators on the gene and protein level by TE and its main ingredient betulin, we firstly studied the impact on NF-κB-DNA binding in an electrophoretic mobility shift assay (EMSA). However, no increased NF-κB DNA binding activity was observed when human primary keratinocytes were incubated either with 1 µg/mL TE or 0.87 µg/mL betulin ( = 1.96 µM) for different time points in contrast to TNF-α which served as positive control (Fig. S4). Betulinic acid (0.04 µg/mL  = 0.08 µM) and lupeol (0.04 µg/mL  = 0.09 µM) were also inactive under these conditions (data not shown).

**Figure 6:**
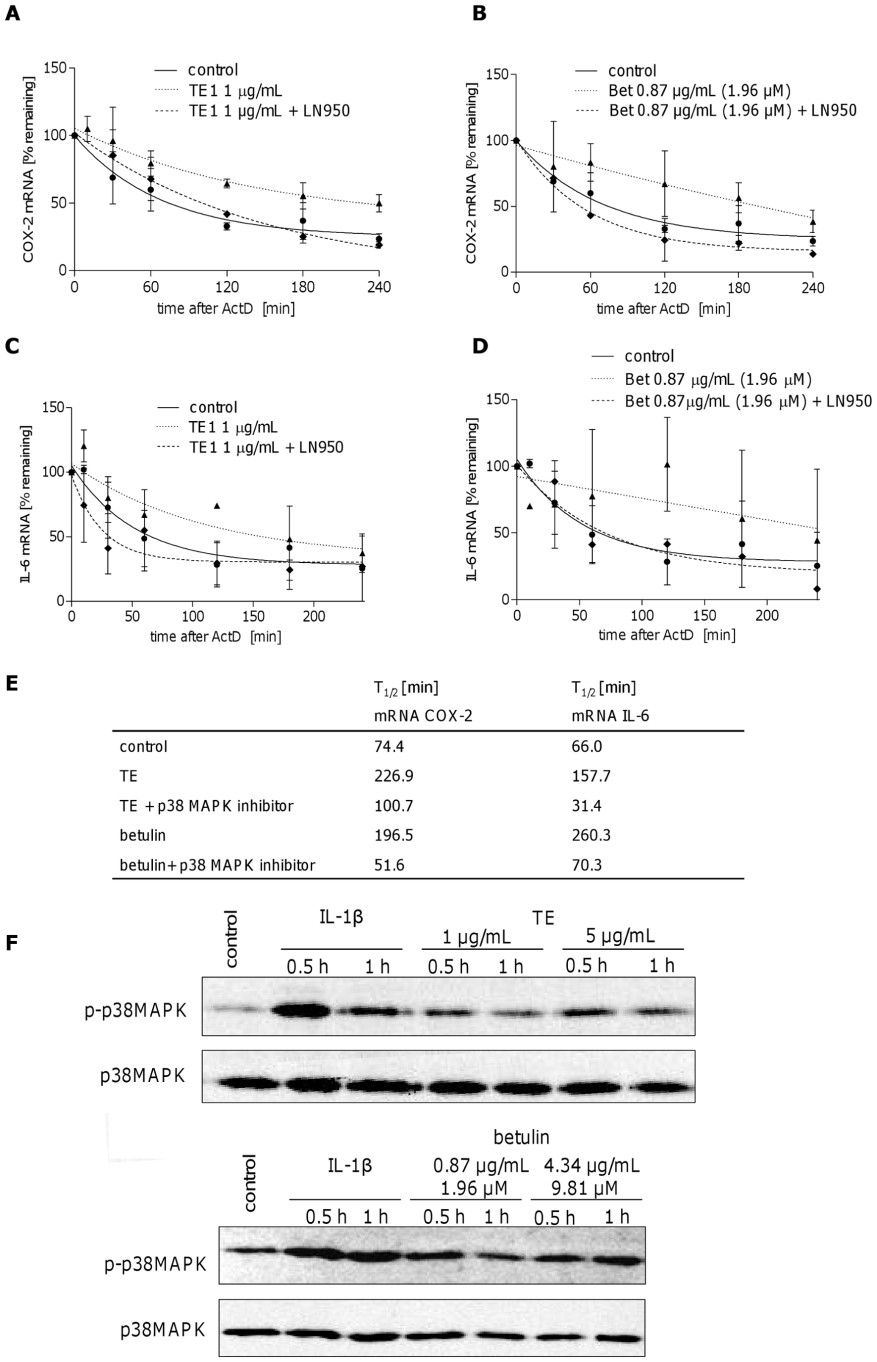
TE and betulin increase the mRNA half-life time of COX-2 (A, B) and IL-6 (C, D) by modulating RNA stability involving p38 MAPK. Primary human keratinocytes were treated either with TE (1 µg/mL) (A, C) or betulin (0.87 µg/mL) (B, D) for 24 h or left untreated followed by ActD (5 µg/mL) for the indicated times with or without the p38 MAPK inhibitor LN950 (100 nM). COX-2 and IL-6 mRNA levels were quantified by qRT-PCR. Results are expressed as % of initial (0 h) mRNA, decay curves are applied and RNA half-life times were calculated (E). Values represent means of at least three independent experiments ± s.d. (F) TE and betulin lead to increased phospho-p38 MAPK levels in Western blot. Primary human keratinocytes were treated either with TE (1 and 5 µg/mL) or betulin (0.87 and 4.34 µg/mL, which is in 1 and 5 µg/mL TE, respectively) for 0.5 and 1 h. IL-1β (20 ng/mL) was used as a positive control. Phosphorylated p38 MAPK (p-p38 MAPK) and total p38 MAPK levels were analyzed. The result of the Western blot was reproduced and one representative Western blot is shown.

Increased mRNA levels of cytokines, chemokines and other pro-inflammatory mediators may also be due to increased stability of mRNA as it has been shown for IL-6, COX-2 and IL-8 [22]–[24]. We therefore exemplarily studied the effect of TE and betulin on IL-6 and COX-2 mRNA stabilization. At first the half-life time of IL-6 and COX-2 mRNA were measured in the presence of actinomycin D (ActD). Figure 6 shows the decay of COX-2 (A) and IL-6 mRNA (C) over time and a half-life time of 74.4 min for COX-2 and 66.0 min for IL-6 calculated by one phase decay non linear regression (Fig. 6E). Treatment of human keratinocytes with TE (1 µg/mL) and betulin (0.87 µg/mL) for 24 h prolongs the half-life time of COX-2 mRNA to 226.9 min by TE and 196.5 min by betulin (Fig. 6A and B). mRNA of IL-6 was also stabilized and resulted in a T_1/2_ of 157.7 min for TE and 260.3 min for betulin (Fig. 6C, D and E).

### p38 MAPK and HuR are involved in the mRNA stabilizing effect of TE and betulin

Since p38 MAPK is known to be primarily involved in COX-2 and IL-6 mRNA stability [24]–[26] we next studied the potential involvement of p38 MAPK in TE and betulin enhanced mRNA stability. Therefore, human primary keratinocytes were treated with TE or betulin and subsequently with the p38 MAPK inhibitor LN950 and ActD and the half-life times for COX-2 and IL-6 mRNA were determined, respectively. Combined treatment of p38 MAPK inhibitor and TE substantially decreased T_1/2_ of COX-2 mRNA to 100.7 min, even though not to the level of the control. Combination of p38 MAPK inhibitor with betulin even led to a lower T_1/2_ as compared to the control (51.6 min). Concerning IL-6, the mRNA half-life time was strongly reduced in the presence of TE and betulin after addition of the p38 MAPK inhibitor. For TE, this resulted in a half-life time lower than the control (31.4 min compared to 66.0 min), whereas the combination with betulin led to a slightly higher T_1/2_ of 70.3 (Fig. 6A–E).

Altogether, these results clearly show that p38 MAPK is involved in TE and betulin-induced COX-2 and IL-6 mRNA stability. These data are supported by increased cellular levels of activated p38 MAPK ( = p-p38) (Fig. 6F) after treatment with TE (1 and 5 µg/mL) and betulin (0.87 and 4.34 µg/mL).

**Figure 7:**
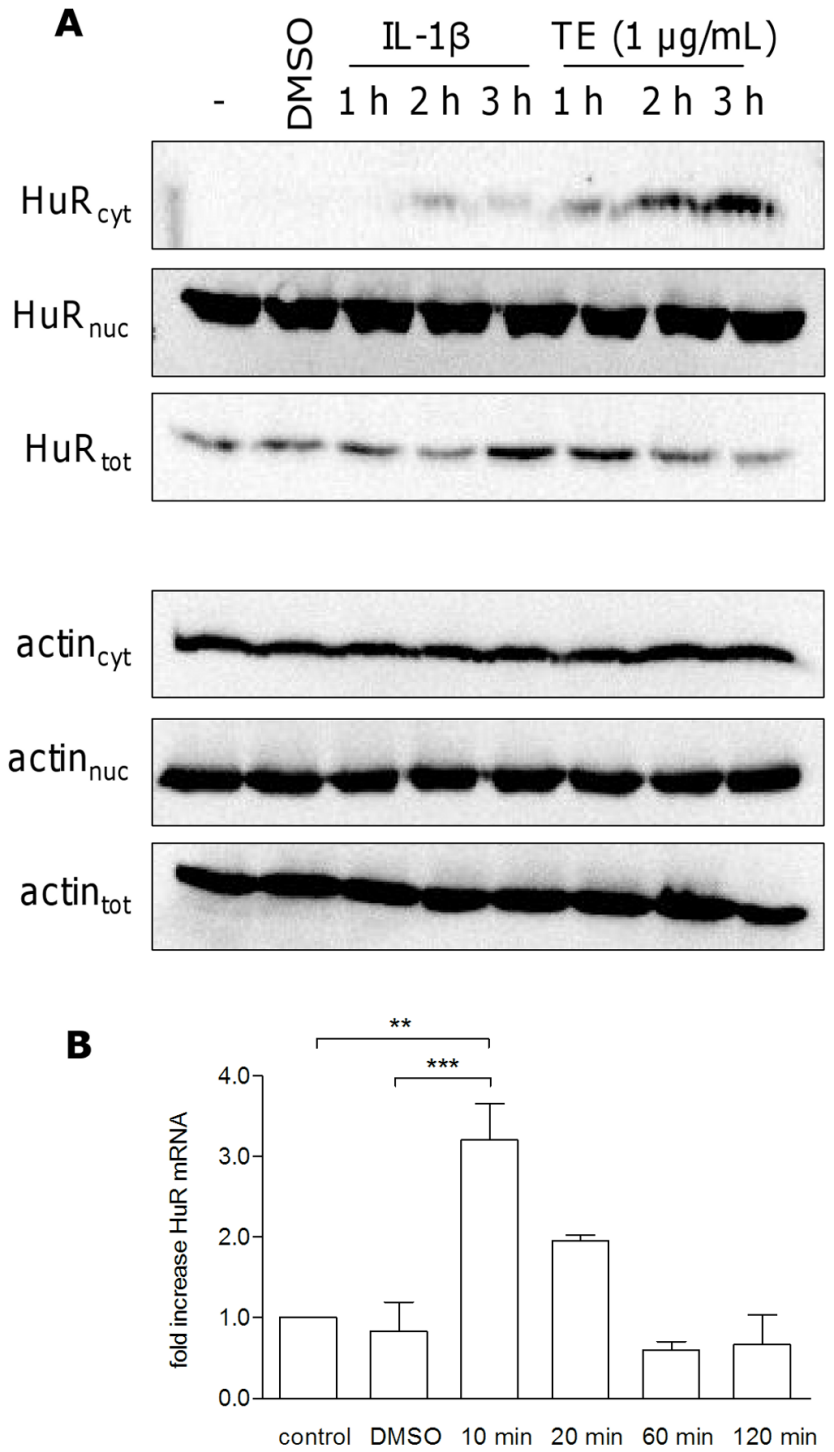
TE increases the amount of cytosolic HuR determined by Western blot analysis. (A). Treatment of human primary keratinocytes with 1 µg/mL TE for 1 to 3 h showed a time dependent increase in cytosolic HuR. IL-1β (20 ng/mL) was used as a positive control, the hyphen indicates untreated cells. Actin was used as a loading control. The result of the Western blot was reproduced and one representative Western blot is shown. (B) TE increases HuR mRNA analysed by qRT-PCR. Primary human keratinocytes were treated with TE (1 µg/mL) for various times. Values represent means of at least two independent experiments ± SEM. **p<0.01 and ***p<0.001 versus control or DMSO.

Subsequently, the effect on HuR, a mRNA stabilizing factor which is known to take part in the posttranscriptional regulation of genes with AU-rich elements (ARE) present in their 3′-untranslated regions [27], was studied. The genes for COX-2 and IL-6 also have this feature and a participation of HuR in COX-2 mRNA stabilization was previously demonstrated [28]. Therefore, the influence on HuR translocation from the nucleus into the cytosol, where it exerts its stabilizing function, was exemplarily assessed with TE by Western blotting. Figure 7A shows that 1 µg/mL TE increased cytosolic localization of HuR. IL-1β (20 ng/mL) was used as control due to its influence on cytoplasmic HuR [29]. Whereas levels of nuclear HuR seem to remain unchanged, total HuR was slightly upregulated after IL-1β (3 h) and TE (1 h). To explain the missing decrease of nuclear HuR levels, qRT-PCR was performed to study whether TE upregulated HuR gene expression. Indeed, TE rapidly enhanced mRNA of HuR after 10 min, which rapidly decreased after 20 min (Fig. 7B). However, the fold increase was low, so that it is questionable that the missing change in the nuclear HuR levels can be solely explained by the new synthesis of HuR. It has to be considered that the change in cytosolic HuR levels may be too small to have an impact on nuclear levels.

### TE activates the transcription factor STAT3

**Figure 8:**
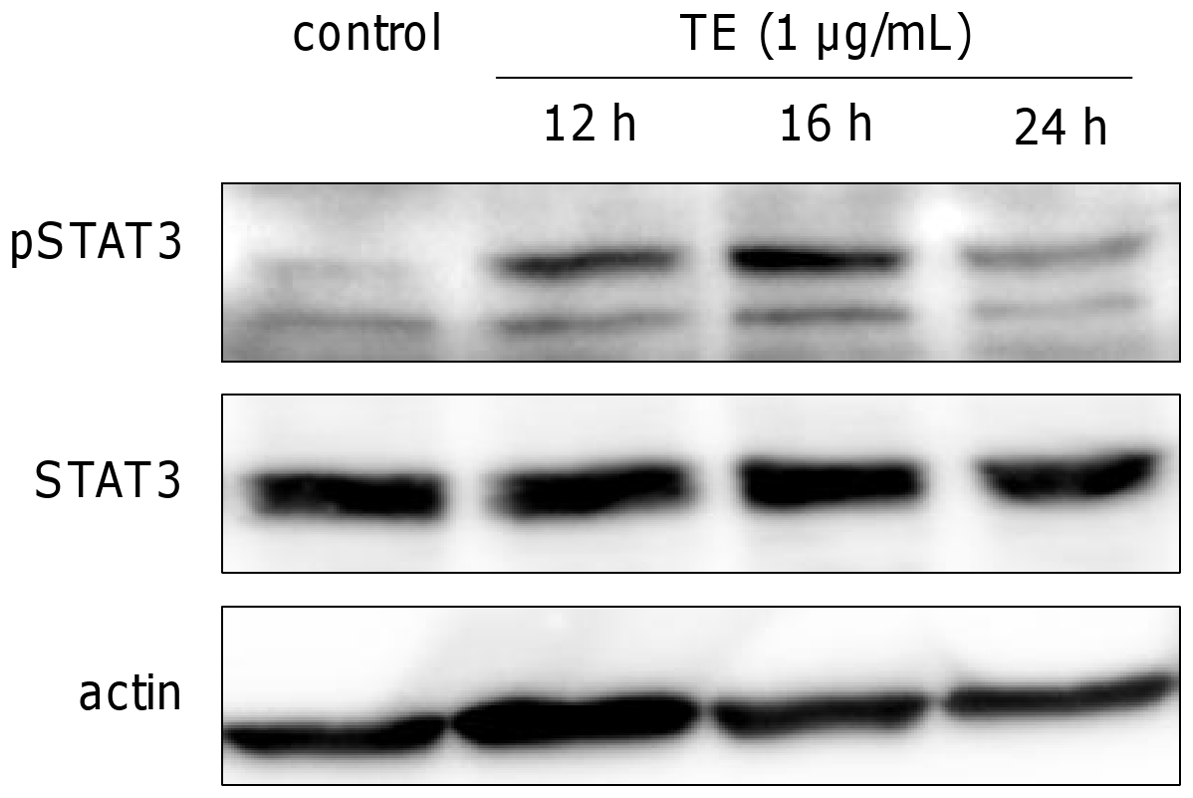
TE (1 µg/mL) induces phosphorylation of the transcription factor STAT3 ( =  pSTAT3). The amount of total STAT3 and actin served as loading controls. The result of the Western blot was reproduced and one representative Western blot is shown.

As mentioned above, treatment of human primary keratinocytes with TE increases IL-6 production (Fig. 4 and 5). This cytokine is an important activator of the transcription factor signal transducer and activator of transcription (STAT3) which plays an essential role in the proliferation and migration of keratinocytes [30]. To study the effect of TE- induced IL-6 on STAT3 activation a Western Blot was performed after different time points. Increase of STAT3 phosphorylation was observed after 12 h and 16 h (Fig. 8).

### TE and betulin do not increase proliferation in primary human keratinocytes and in the WHM

Because of the putative effect of STAT3 on proliferation, we investigated the effect of 1 µg/mL and 5 µg/mL TE, as well as the respective concentrations of betulin, on proliferation of primary human keratinocytes. We did not observe any proliferative effect with TE, betulin or with lupeol and betulinic acid, which were also included in the study (Fig. S5A). This was also confirmed in the WHM (Fig. S5B). There was no significant influence on proliferation in all parts of the WHM even though there was a trend of decreased number of proliferative cells after treatment with 10 µg/mL TE and betulin (8.69 µg/mL).

### TE enhances migration of primary human keratinocytes in the scratch assay

**Figure 9:**
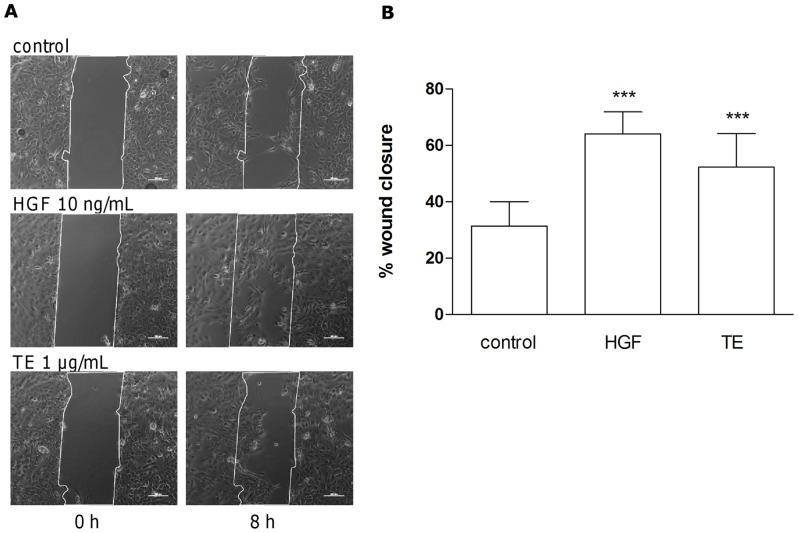
TE enhances migration of primary human keratinocytes in the scratch assay. Cells were incubated with 1 µg/mL TE, 10 ng/mL HGF or treated with DMSO, as control. Images were taken immediately after wounding (0 h) and after 8 h incubation (A). The scale bar is of 100 µm width. Representative images of repeated experiments are shown. (B) Closed area in % after 8 h (1 µg/mL TE, 10 ng/mL HGF or untreated control) compared to the scratch area at time point zero (0 h). Values represent mean of closed areas of 4 independent experiments ± s.d. ***p<0.001 versus control.

Reepithelialization comprises proliferation and migration. We observed increased reepithelialization in the TE-treated WHM (Fig. 1) but no proliferative effects (Fig. S5). Hence we studied the impact on migration in scratch assay experiments with primary human keratinocytes using ibidi® culture inserts. Cells were treated with 1 µg/mL TE and 10 ng/mL hepatocyte growth factor (HGF), as a positive control [31] and microscopic images were taken immediately after wounding (t = 0 h) and during an incubation time of up to 24 h. Both stimuli showed a significant increase of closure of the scratch (64% ±15 for HGF and 52% ±24 for TE) compared to untreated cells (31%±17) 8 h after wounding (Fig 9A and B).

### TE and its triterpenes affect the actin cytoskeleton

The reorganization of the actin cytoskeleton in terms of controlled polymerization and depolymerization is involved in cell motility. It provides a driving force for migration of cells, such as keratinocytes, at the wound edge, which is necessary for wound closure [32]. Filopodia, lamellipodia and stress fibers are different kinds of actin structures, which contribute to cell migration.

**Figure 10:**
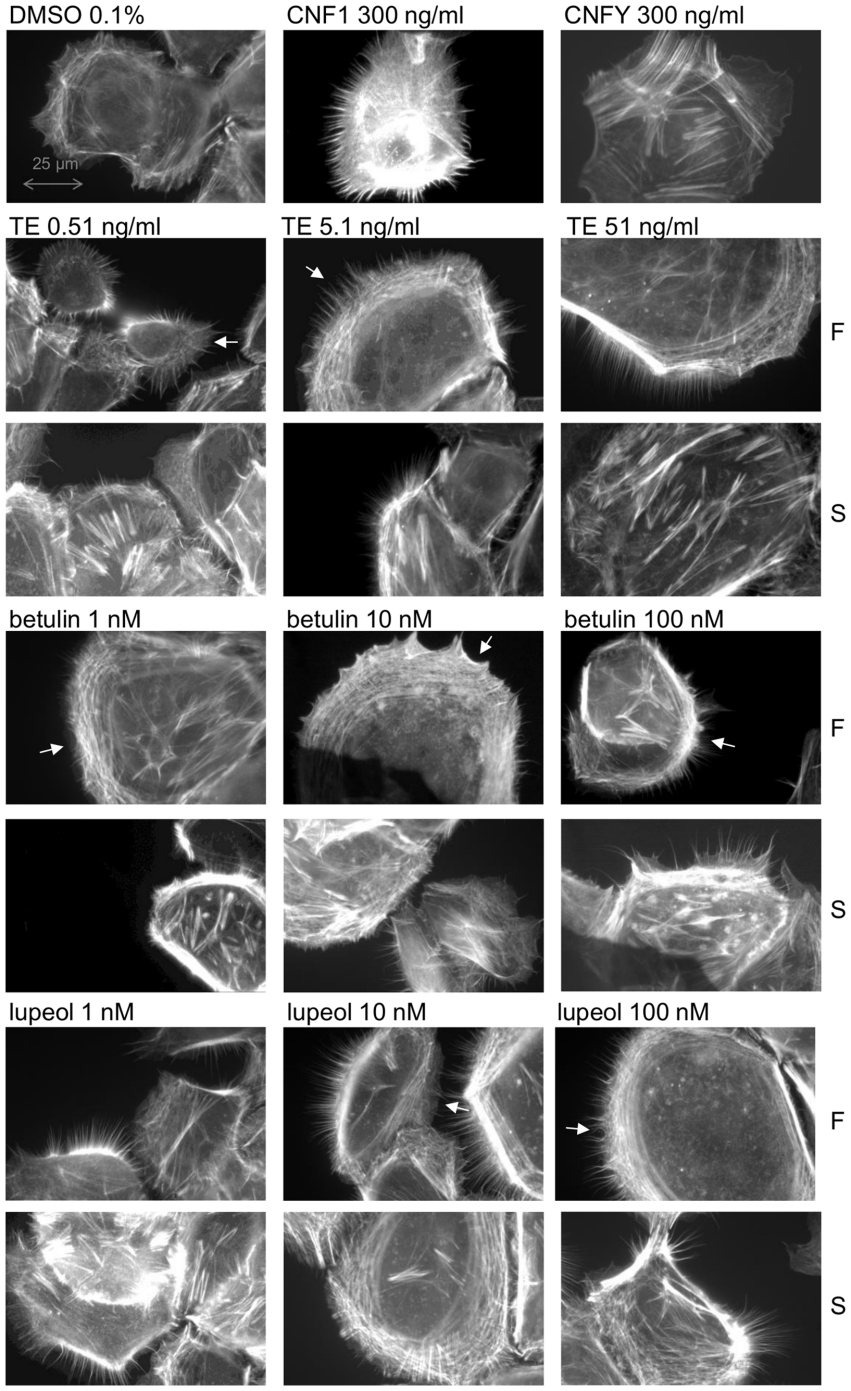
TE, betulin and lupeol influence the actin cytoskeleton of primary human keratinocytes. Cells were incubated on glass coverslips for 2/mL TE and 1, 10 and 100 nM betulin and lupeol, respectively. The actin cytoskeleton was stained with phalloidin-rhodamine. 0.1% (v/v) DMSO was used as solvent control and CNF1 and CNFY as positive controls. Rows labeled with F show the impact on filopodia and lamellipodia and S the impact on stress fiber formation. A white arrow indicates the leading edge of the cell. Representative pictures of repeated experiments (n = 5) are shown.

To study whether TE and its main triterpenes influence the actin cytoskeleton, primary human keratinocytes were treated with different concentrations of TE and the respective isolated triterpenes for two hours and stained with fluorophore labelled phalloidin. Subconfluent cells were used, as they phenotypically and biochemically resemble the active keratinocytes from the basal layer at the wound margin migrating on the newly formed granulation tissue until recovering the injured skin [33], [34]. Treatment with TE at concentrations of 0.5 and 1 µg/mL induced formation of stress fibers (S), filopodia and lamellipodia (F) (Fig. S6). Interestingly, even lower concentrations of TE (0.51, 5.1 and 51 ng/mL) induced the formation of filopodia and lamellipodia (F) as well as stress fibers (S) to a greater extent compared to the higher concentrations (Fig. 10). Long, subtle and spiky filopodia and a very dense, stripe-like actin meshwork (lamellipodia) were observed at the leading edge of the cell compared to the solvent control DMSO, where only few short filopodia were visible. As positive control for the formation of filopodia and lamellipodia, cytotoxic necrotizing factor 1 from *Escherichia coli* (CNF1) was used which directly activates Rac and Cdc42. For the induction of stress fibers, CNFY (cytotoxic necrotizing factor from *Yersinia pseudertuberculosis*) was applied which selectively activates RhoA [35].

The single triterpenes of birch bark extract behaved differently. Betulin was tested at concentrations of 1, 10 and 100 nM, corresponding to the amount of this triterpene in 0.51, 5.1 and 51 ng/mL of TE. Lupeol, betulinic acid, oleanolic acid and erythrodiol were tested at concentrations of 1, 10 and 100 nM, due to comparability reasons to betulin. Betulin and lupeol strongly polarized the cells by formation of broad lamellipodia and spiky filopodia at the leading edge (indicated by white arrows) even at the lowest nanomolar concentration (Fig. 10). Stress fiber assembly within the cell body, particularly at the lowest concentration (1 nM), was also increased by both triterpenes. No effect could be observed for betulinic acid and oleanolic acid at any tested concentrations, whereas erythrodiol (1–100 nM) increased the formation of stress fibers, filopodia and lamellipodia compared to the solvent control DMSO (see Fig. S6).

### TE, betulin and lupeol activate Rho GTPases involved in regulation of the actin cytoskeleton

Members of the Rho family of GTPases, especially RhoA, Rac1 and Cdc42 are essentially involved in the regulation of the actin cytoskeleton [36]–[39]. Activation of Cdc42 polarizes the cell [40], leads to the formation of filopodia [41] and also affects lamellipodia [42], whereas activation of Rac results in lamellipodia extension [37], [42], [43], which is the driving force of cell migration. The formation of actin-myosin containing stress fibers, which contract the cell body, as well as the stress-fiber associated focal adhesions, the anchorage point to the substrate, is finally mediated by RhoA [43].

**Figure 11:**
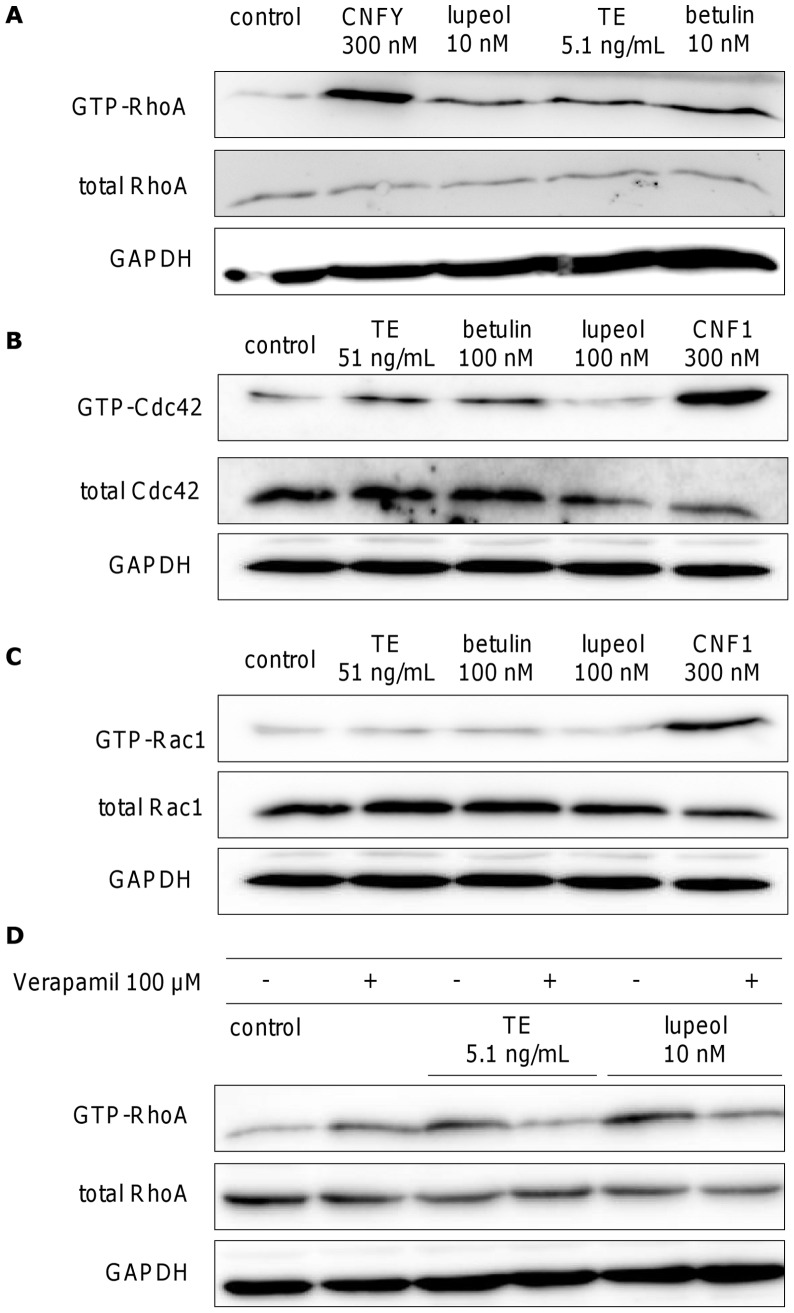
Influence of TE (5.1 or 51 ng/mL), betulin (10 or 100 nM) and lupeol (10 or 100 nM) on the activity of the Rho GTP-binding proteins RhoA (A), Cdc42 (B) and Rac1 (C) after 3 h incubation measured by pulldown experiments in primary human keratinocytes. CNFY and CNF1 were used as positive controls. (D) Influence of the calcium channel blocker verapamil (100 µM) on the activation of RhoA. Verapamil was added 10 min prior to treatment with TE (5.1 ng/mL) and lupeol (10 nM). Each experiment was reproduced and a representative Western blot is shown.

Due to the strong effects of TE, betulin and lupeol on different cytoskeletal actin structures (Fig. 10 and S6) we examined their influence on the above mentioned Rho GTPases using exemplarily 5.1 ng/mL of TE, 10 nM betulin (corresponding concentration in 5.1 ng/mL TE), and 10 nM lupeol. Figure 11A shows the effect of TE, betulin and lupeol on RhoA in primary human keratinocytes assessed by pulldown experiments and subsequent Western blot analysis. The active form of RhoA, GTP-RhoA, was precipitated with rhotekin-loaded beads after 3 h of incubation with the respective compounds. TE, betulin and lupeol, were able to slightly activate RhoA. Cytotoxic necrotizing factor from *Escherichia coli* (CNFY) (300 nM), which constitutively activates RhoA by deamination of glutamine 63, that arrests RhoA in the active, GTP-bound state, was used as positive control [44]. Slight effects on Cdc42 were only observed at a ten-fold higher concentration of TE (51 ng/mL) and betulin (100 nM), but not for lupeol (100 nM) (Fig. 11B). CNF1 (300 nm) which also deaminates Cdc42 and Rac1 at glutamine 61 was used as positive control [45]. There was no clear activation of Rac1 with TE and the triterpenes (Fig. 11C).

Calcium is a multifaceted second messenger connecting receptor activation to downstream signaling pathways and is also involved in the regulation of Rho GTPases [46]–[48]. Since TE has been shown to increase intracellular calcium levels in primary human keratinocytes [49], we exemplarily examined the role of calcium in TE and lupeol mediated RhoA activation by using verapamil, a blocker of voltage dependent calcium channels [50]. Primary human keratinocytes were pretreated with 100 µM verapamil 10 minutes prior to incubation with either TE (5.1 ng/mL) or lupeol (10 nM) (see Fig. 11). Reduction of the activation of RhoA by TE and lupeol, when using the inhibitor, shows that calcium is likely involved in RhoA activation.

## Discussion

The aim of this study was to elucidate the underlying molecular mechanism of the clinically proven wound healing effect of a birch bark extract (TE), which consists mainly of different pentacyclic triterpenes. We used primary human keratinocytes as a model, because they are the main cell population of the epidermis and are essential for the rapid coverage of the wound with a neo-epidermis. We demonstrate that TE influenced the inflammatory phase as well as the new tissue formation phase of the wound healing process. Regarding pro-inflammatory effects only betulin, the main constituent of TE, was shown to be responsible, whereas an influence on cell migration, measured by rearrangement of the actin cytoskeleton, could additionally be observed for lupeol and erythrodiol. These processes may contribute to the improvement of the epidermal regeneration and acceleration of the repair of the epidermal barrier function by TE proven in the porcine *ex-vivo* model. Kinetics of wound healing have not been evaluated in the *ex-vivo* model yet, but would be an interesting task in the future.

Related to the inflammatory phase TE and betulin led to a time and concentration dependent increase of COX-2 and IL-6 mRNA in primary human keratinocytes. This result was confirmed in the porcine *ex-vivo* wound healing model, and on the protein level of primary human keratinocytes. The importance of COX-2 in the wound healing process was shown by Futagami et al., who observed delayed reepithelialization of rat skin wounds after application of a COX-2 specific inhibitor [17]. COX-2 knock-out mice exhibited diminished tensile strength after dermal wounding compared to wild type [51]. However, the role of COX-2 in wound healing has not yet been doubtlessly clarified. Several studies demonstrated that COX-2 inhibition had no or only a minor negative effect on wound healing [52]–[54]. Given that the birch bark extract had already shown promising wound healing properties in patients [14] the observed slight increase of COX-2 expression after TE treatment may be beneficial in wound healing.

The importance of IL-6 in wound healing was demonstrated in experiments with IL-6 knock-out mice, which displayed impaired wound healing resulting in decelerated reepithelialization, diminished angiogenesis, collagen deposition and leucocyte infiltration [18], [55]. IL-6 was also evidenced to be involved in epidermal barrier repair, as topical application of IL-6 enhanced epidermal barrier repair in wild-type mice, whereas inhibition of the agonist IL-6/sIL-6 receptor complex, delayed barrier repair in wild mice [56]. We also observed an improved skin barrier function after treatment with TE and betulin, which may be linked to increased levels of IL-6. Pantothenate, a common self-medication for wound healing, also showed an upregulation of IL-6 in cultured human fibroblasts [57]. This was discussed by the authors as a contribution to wound healing properties of pantothenate. IL-6 is also a well-known activator of the JAK/STAT signaling cascade [58]. STAT3 activation commonly results in expression of genes both related to migration and proliferation [59]. However, in case of the birch bark extract, only cell migration seems to be affected, since we detected no increase in the number of proliferative cells. Beside IL-6, the Rho GTPase RhoA, which was slightly activated by TE, could additionally contribute to the observed STAT3 activation as previously shown by Reipschläger et al. [60].

Further studies were performed to elucidate the mechanism of increased mRNA levels of COX-2 and IL-6 under TE and betulin treatment. The transcription factor NF-κB, which is a potent inducer of many pro-inflammatory genes [21], was not involved. Instead, we found a prolonged mRNA half-life. p38 MAPK has been shown to participate in mRNA expression and stability of many proinflammatory mediators including COX-2 and IL-6 [24]–[27]. We demonstrated its involvement using a specific p38 MAPK inhibitor, which reduced the prolonged half-life time. Accordingly, TE and betulin slightly increased the amount of phosphorylated p38 MAPK as shown by Western blot analysis in primary human keratinocytes.

p38 MAPK functions by activating or inhibiting mRNA stability or destability factors like HuR and TTP [61], [62]. HuR exerts its stabilizing function only when localized in the cytosol [63]. Our data suggest that TE led to an increase of the cytosolic HuR fraction. Since p38 MAPK is known to induce the nuclear export of this factor [64], [65], the observed HuR accumulation may be the result of p38 MAPK activation. Nucleo-cytoplasmatic shuttling of HuR normally implicates a decrease in nuclear HuR levels, which we could not observe. The missing reduction in nuclear HuR can possibly be explained by rapid de novo synthesis, as we measured an increase in HuR mRNA level. However, it cannot be excluded that the differences in protein level are too small to be detectable by Western blot analysis.

In addition to COX-2 and IL-6, IL-8 and TNF-α were shown to be upregulated on gene and protein level by TE and betulin in primary human keratinocytes. By simultaneous measurement of different pro-inflammatory cytokines, chemokines and growth factors with the Bio-Plex® cytokine assay we achieved a more comprehensive overview about other affected mediators. Notably, among the mediators especially the tested chemokines (IL-8, IP-10, MIP-1β, and RANTES) were remarkably upregulated by TE and betulin treatment in keratinocytes. The chemokine IL-8 is known to be significantly upregulated in healing skin wounds and can be found in the wound fluid [20], [66], where it acts as a potent chemoattractant recruiting and activating inflammatory cells [67], [68]. Topical application of IL-8 has been shown to enhance wound healing through accelerated reepithelialization [20].

In summary, our results show that TE and betulin upregulates various mediators being involved in the inflammatory phase of the wound healing process. Pro-inflammatory effects of some of the investigated triterpenes have already been previously reported. Similar to our results, Zdzisinska et al. demonstrated a moderate induction of TNF-α by betulin in primary human whole blood cell cultures [69]. Betulinic acid was shown to induce TNF-α production in mouse macrophages [70]. However, it has also been demonstrated that betulin, lupeol and betulinic acid exert potent anti-inflammatory effects *in-vivo* and *in-vitro* [71]–[73]. Thus, it seems to strongly depend on the concentration, time of incubation, examined cell or tissue type (primary or cancer cells), and the experimental setup (stimulated vs. unstimulated cell systems) [74], whether these triterpenes exhibit inflammatory or anti-inflammatory effects.

Whereas a temporary inflammation is necessary for the wound healing process, an excessive and prolonged inflammatory phase is deleterious for proper wound healing and leads to chronic wounds. Hence, a termination of the inflammatory response is indispensable [1], [3], [6], [75]. In the WHM we clearly demonstrated that the increase in COX-2 and IL-6 mRNA was only temporary. Moreover, COX-2 is not only involved in pro-inflammatory effects, in later stages of wound healing the enzyme promotes the formation of anti-inflammatory prostaglandins, e.g. PGD_2_ and PGJ_2_ derivatives, which contribute to resolution of inflammation [76], [77]. Additionally, TGF-β, which was slightly upregulated by TE, can contribute to a termination of the inflammatory phase by induction of IL-1ra and reduction of TNF-α, IL-8, MCP-1 und MIP-1α [78]. Taken together, we assume that TE and betulin enhance the beginning of the inflammatory phase of wound healing by a transient upregulation of pro-inflammatory mediators, which does not result in a prolonged inflammation.

Furthermore, our studies provide evidence that birch bark extract influences the second stage of wound healing, the new tissue formation phase, by increasing cell migration of primary human keratinocytes shown in a scratch assay experiment. Migration strongly depends on morphological polarization of the cells and includes membrane protrusions at the leading edge of the cell with formation of new attachments to the substrate, contraction of the cell body and finally release of the rear [79]. These processes require a coordinated interaction of cytoskeletal elements, especially the assembly and disassembly of actin filaments, which lead to the formation of distinct cellular structures such as filopodia, lamellipodia and stress fibers [80]–[82]. Compounds, which directly or indirectly affect these actin structures, are interesting in context of supporting and enhancing wound healing processes. We clearly showed that TE in micromolar and even in nanomolar concentrations strongly influenced the actin cytoskeleton resulting in polarization of the cells, formation of lamellipodia and filopodia at the leading edge and stress fibers in the cell body. Formation of stabilized filopodia and stress fibers was additionally confirmed by visualizing vinculin with fluorescence labeled antibodies (data not shown). This protein accumulates in newly formed focal complexes in filopodia and in focal adhesions in the whole cell body [83], [84]. The influence on the actin cytoskeleton in nanomolar concentrations could be confirmed with the isolated triterpenes betulin, lupeol and erythrodiol. Notably, in mouse melanoma cells, Hata et al. observed just the opposite effect, that means a disassembly of stress fibers under lupeol treatment, which suppressed cell migration at concentrations of 1 to 20 µM [85]. Martin et al. also showed a disassembly of stress fibers for erythrodiol (25 to 50 µM) in a human astrocytoma cell line [86]. These contrasting results may be explained by the fact that cancer cell lines were used by these groups. In accordance with this conclusion. Martin et al. observed disrupted stress fibers in a human astrocytoma cell line after oleanolic acid treatment [87], whereas the same compound showed no alteration of cytoskeletal structures in primary human dermal fibroblasts [88], supporting our findings in primary keratinocytes.

Stress fiber formation is regulated by activation of the Rho GTPase RhoA [41], [43]. Consequently, we could demonstrate an increase in GTP-RhoA by TE, betulin and lupeol, indicating that activation of RhoA might contribute to the observed stress fiber formation. It is known that calcium is involved in the regulation of Rho GTPases and other downstream players, like actin-binding proteins, which directly control actin polymerization and depolymerization [46]–[48], [89], [90]. Accordingly, we could also demonstrate the involvement of calcium in TE- and lupeol-mediated RhoA activation using verapamil, a blocker of voltage dependent L-type calcium channels [50], as a significant reduction of GTP-bound RhoA by verapamil treatment was observed. An increase in intracellular calcium using a comparable birch bark extract was also previously reported by Woelfle [49]. This group additionally demonstrated an augmented expression of TRPC6 (transient receptor potential superfamily of cation channels 6) after stimulation with their birch bark extract, and discussed that this effect may be due to the observed calcium influx. Considering both results on Ca^2+^ influx, it can be concluded that TRPC6 may not be exclusively and entirely responsible for calcium influx, but that activation of L-type calcium channels can be generated by TRPC6 mediated membrane depolarization, as reported by Soboloff et al. [91].

Changes in the actin cytoskeleton and Rho GTPase activation are closely related to mitogen activated protein kinase pathways [92]. Thus, Rho GTPases can lead to phosphorylation of p38 MAPK via influencing upstream kinases [38]. Therefore, the activation of p38 by TE and betulin may not only affect mRNA stabilization, but also be involved in the observed assembly and disassembly of actin filaments. TE and betulin also activated the Rho GTPase Cdc42, which is known to stimulate filopodia formation [41], [43], but here higher concentrations were required compared to RhoA activation. Interestingly, lupeol, which also increased the amount of filopodia, did not activate Cdc42. Additionally, no activation of Rac1 by TE, betulin or lupeol was observed, despite increased formation of lamellipodia. These observations may be explained by spatial or temporary activation or the involvement of other Rho GTPases like Rif [93].

In summary our results contribute to the understanding of the molecular mechanism of the clinically proven wound healing effect of birch bark. Our results, together with the proven efficacy, identify birch bark as the first medical plant with a high potential to improve wound healing, a field which urgently needs effective remedies. Moreover, birch bark is a successful example that traditional medicinal plants can become rational drugs.

## Materials and Methods

Foreskin samples used for keratinocyte culture were obtained following medical circumcisions. Its use was approved by the ethics committee of the Aerztekammer Hamburg, Germany (060900) and Freiburg, Germany (45/03). The ethics committee waived the need for consent, respectively. Porcine skin was obtained from a local slaughterhouse. The animals were slaughtered for human consumption and the ears were harvested following slaughtering with the permission of the slaughterhouse (Hoose, Hammor).

### Tissues, antibodies, reagents and cell culture consumables

The birch bark extract (TE), betulin, lupeol, betulinic acid, erythrodiol and oleanolic acid were received from Birken AG, Niefern-Öschelbronn, Germany. Porcine skin was obtained from a local slaughterhouse. All the pigs were of the same age (6 months) and race (crossbred Yorkshire/Deutsches Edelschwein). The number of pigs or keratinocyte samples used in the various experiments is denoted in the respective figure legends.

NF-κB oligonucleotide: Promega, Mannheim, Germany; [γ33P]ATP: Hartmann Analytic, Braunschweig, Germany; T4 polynucleotide kinase: New England Biolabs, Frankfurt, Germany; Antibiotic-Antimycotic® 100×, phalloidin-rhodamine, Prolong Gold® Antifade reagent, Keratinocyte SFM® and supplements (human recombinant EGF, bovine pituitary extract), FCS, Trypsin/EDTA 0,05%/0,02%: Life Technologies, Darmstadt, Germany; Ciprobay®: Bayer, Leverkusen, Germany; LightCycler® 480 Probes Master, penicillin-streptomycin, poly(dI-dC), Complete® EDTA free protease inhibitor cocktail, PhosStop®, 0.45 μm PVDF membrane: Roche, Mannheim, Germany; Quantikine® Human IL-6 and IL-8 ELISA, Parameter^TM^ PGE_2_ ELISA: R&D Systems, Minneapolis, USA; RNeasy® Plus Mini Kit and QuantiTect® Reverse Transcription Kit: Qiagen, Hilden, Germany; primer and probes for quantitative RT-PCR, HGF, phosphatase inhibitor cocktail 2: Sigma, Steinheim, German; ActD: Enzo Life Sciences, Lörrach, Germany; LN950: Prof. S. Laufer, University of Tübingen, Germany; Bio-Plex® Cytokine Assay and Bradford Quick Start Dye: Bio-Rad Laboratories, Munich, Germany; ibidi culture inserts with μ-dishes: ibidi, Munich, Germany; DS-QiMc: Nikon Instruments Inc., Tokyo, Japan; GST-Rhotekin and GST-PAK (immobilized to glutathione-sepharose beads, Pharmacia Biotech, Cambridge, USA): Prof. G. Schmidt, Albert-Ludiwgs-University Freiburg, Germany; HRP juice: pjk, Kleinblittersdorf, Germany; pSTAT3, STAT3, p38 and pp38 MAPK antibody: Cell Signaling, Danvers, USA; HuR, RhoA and Rac1 antibody: Santa Cruz, USA; Cdc42 antibody: Millipore, Bedford, USA; COX-2 antibody: Cayman, Chemical Company, USA; anti rabbit and anti mouse antibody: Jackson Immuno Research, Newmarket, UK; Ki67-antibody: MIB; Dako, Hamburg, Germany.

### Isolation and cultivation of primary normal human keratinocytes (NHK)

Normal human skin keratinocytes (NHK) from foreskin explants were isolated and cultivated as described in Protocol S1.

### *Ex-vivo* porcine wound healing model and reepithelialization (granted patent DE 10317400)

Pig ears were removed directly after slaughtering, delivered immediately to the laboratory, washed, and disinfected. Subsequently, punch biopsies (ø 6 mm diameter) were taken from the plicae of the ears and fat, subcutis and parts of the dermis were removed. Wounds were formed by removing epidermis and upper dermis from the central area of the biopsies (ø 3 mm). Finally, the models were placed dermis down on gauze in culture dishes and incubated air-liquid interface with Dulbecco's modified Eagle's medium supplemented with hydrocortisone, 2% fetal calf serum, penicillin, and streptomycin at 37°C with 5% CO_2_. The substances were applied immediately after wounding, for substances diluted in PBS application was repeated after 24 h. After 48 h, models were shock frozen in liquid nitrogen and stored at −80°C. Subsequently, cryostat sections were gained from the central parts of the wound healing models and stained with hematoxylin and eosin [94]. Reepithelialization was evaluated by measuring the distance between the wound margin to the tip of the regenerated epidermis with an axiophot II Zeiss microscope and the measurement tool of Openlab 5.0.2 software (Improvision, Coventry, UK). Means of left and right wound margins were calculated. Pigs with completely closed wounds in controls were excluded because an improvement of reepithelialization compared to PBS could not be unambiguously evaluated in these wounds.

### *Ex-vivo* barrier assay

The WHM was established as described above. For the investigation of the early effect of the substances on barrier regeneration they were applied directly after wound healing. After 4 days the upper surface of the models was incubated for 5 min in methanol, for 5 min in PBS and for 15 min in toluidine blue (0.1% in PBS). Excessive dye was removed and models were shock frozen. For the investigation of late effects on barrier regeneration the substances were applied 72 h after wounding and toluidine blue staining was performed after another 24 h of incubation. Subsequently sections of the middle of the models were generated and the distance of regenerated epidermis without dye penetration starting from the wound margin was measured with an axiophot II Zeiss microscope and Openlab 5.0.2 software (Improvision; Coventry, UK).

### RNA isolation, cDNA synthesis and quantitative Real-Time PCR

Total RNA from normal human keratinocytes was isolated using the RNeasy® Plus Mini Kit from Qiagen and converted to single strand cDNA using the QuantiTect® Reverse Transcription Kit according to the manufactures instructions. The analysis of mRNA expression profiles was performed with quantitative real time PCR as described in detail in Protocol S1.

### Determination of the mRNA half-life time

For determination of the mRNA half-life time of IL-6 and COX-2 primary human keratinocytes were incubated with the transcription inhibitor ActD (5 µg/mL) for the indicated times. Subsequently, total RNA was harvested and IL-6 and COX-2 expression was quantified by qRT-PCR at different time points, respectively. The amount of mRNA without ActD treatment (time point zero) was referred to as 100% and the amount at different time points as percentage of remaining mRNA. From the calculated curves (one phase decay curve using GraphPad Prism 5) the time of 50% mRNA decay was deduced. To measure mRNA stability in response to TE and betulin treatment keratinocytes were stimulated either with TE or betulin in the presence of ActD (5 μg/mL). IL-6 and COX-2 mRNA expression were again quantified by qRT-PCR after the indicated times and calculation was performed as described in Protocol S1. To analyze the influence of p38 MAPK on the half-life time the p38 MAPK inhibitor LN950 (100 nM) was added simultaneously with ActD. Means of three independent experiments are presented.

### IL-6 and IL-8 ELISA

Cells were seeded into 6-well plates at a density of 200 000 cells/well. After 24 h, medium was changed to medium without supplements and incubated for additional 24 h before test compounds were added. Upon incubation for various times, cell culture supernatants were transferred to reaction tubes, centrifuged for 5 min at 10.000 rpm and aliquoted in new reaction tubes. A Quantikine® Human IL-6 and IL-8 ELISA were used according to the manufacturer's instruction, respectively.

### Bio-Plex® Cytokine Assay

To simultaneously determine the influence of the tested compounds on a variety of cytokines, chemokines and growth factors, the Bio-Plex® Human Cytokine Assay was used. After a period of 24 h of incubation supernatants were obtained according to the method described for the IL-6 and IL-8 ELISA. Measurement of the supernatants was performed according to the manufactureŕs instruction.

### Scratch Assay

Monolayer wounding assays were used to evaluate migration of keratinocytes as described before [95]. Briefly, NHK cells were grown to confluence in ibidi μ-dishes containing culture inserts. Cells were incubated in Keratinocyte SFM® medium (with rhEGF, BPE and penicillin-streptomycin) containing 1 μg/mL TE or 10 ng/mL HGF. Live cell imaging migration was recorded in the PFS system on a Nikon Eclipse Ti microscope with a digital sight DS-QiMc (Nikon Instruments Inc., Tokyo, Japan), coupled to an ibidi-heating chamber. The migratory activity was measured by calculating the percentage of closed areas.

### Determination of proliferation in WHM and cultured keratinocytes

Proliferative (Ki67-positive) cells were detected by MIB-1 in WHM by immunofluorescence staining as described previously [96] and in Protocol S1. Proliferation of cultured human keratinocytes was measured with the BdrU-ELISA kit from Roche (Mannheim, Germany) as described in Protocol S1.

### Staining of the actin cytoskeleton

To analyze the effect of TE and the single triterpenes on the actin cytoskeleton, cells were stained with phalloidin-rhodamine and analyzed by fluorescence microscopy as described in Protocol S1.

### Preparation of the protein extracts for Western Blotting and EMSA

Whole cell, cytosolic and nuclear extracts from primary human keratinocytes were prepared as described in detail in Protocol S1.

### Electrophoretic mobility shift assay (EMSA)

Nuclear protein was resolved through non-denaturing 6% polyacrylamide gel electrophoresis and NF-κB-DNA binding was detected according to the Protocol S1.

### Rho-GTPase pulldown experiments

Cell extracts were obtained as described by [60]. Briefly, cells were scraped in lysis buffer containing 10% glycerol, 50 mM Tris pH 7.4, 100 mM NaCl, 1% NP-40, 2 mM MgCl_2_ and 1 mM PMSF. After centrifugation at 4°C, the supernatant was used for determination of total Rho GTPase content and for pulldown experiments. For pulldown analysis of RhoA and Cdc42/Rac1, GST-Rhotekin or GST-PAK immobilized to gluthatione-sepharose beads were incubated with the supernatant for 1 h under gently shaking at 4°C. Subsequently, the beads were washed and separated by centrifugation. The Rho GTPases were subsequently analyzed by immunoblot.

### Immunoblot analysis

The protein concentration was determined by using the Bradford Quick Start Dye according to the manufacturer's instruction. For Western blot analysis, 50 µg of protein was separated by a 10–12% SDS-PAGE and transferred to a 0.2 or 0.45 μm PVDF membrane. After blocking the membrane with 5% milk powder in TBST (TBS buffer containing 0.1% Tween 20) or 3% BSA in TBST for HuR, it was incubated with the respective antibody overnight at 4°C under gently shaking. The proteins were detected by using horseradish peroxidase labeled secondary antibodies and an enhanced chemiluminescence detection system.

Antibody concentrations: pSTAT3 (Tyr705) and STAT3: 1∶1000 in 5% BSA in TBST; pp38 and p38 MAPK: 1∶1000 in 5% BSA in TBST; HuR: 1∶500 in 5% milk in TBST; COX-2: 1∶1000 in 5% milk in TBST; RhoA: 1∶400 in TBST; Rac1: 1∶2000 in TBST; Cdc42: 1∶500 in 3% milk in PBS; anti rabbit: 1∶5000 in 5% BSA in TBST; anti mouse: 1∶5000 in 3% milk in TBST.

### Statistical analysis

Values are expressed as means ± standard deviation (s.d.) or ± standard error of the mean (SEM). Statistical analyses of data sets were performed by using ANOVA followed by Dunnett's post-test. P-values were calculated and p<0.05 was considered significant. Wherever significance has been proven, it is indicated by *p<0.05, **p<0.01 and ***p<0.001.

## Supporting Information

Figure S1 **Quantitative analysis by gas chromatography and structures of triterpenes in birch bark.** A: Quantitative composition of TE analyzed by gas chromatography [15]: betulin (86.85%), lupeol (3.94%), erythrodiol (0.77%), oleanolic acid (0.62%), betulinic acid (3.52%). In brief, 0.2 mg of TE was diluted in 500 µL tetrahydrofuran. This solution was silylated with 100 µL of silylating mixture (Silylating Mixture Fluka III; Buchs, Switzerland) at 80°C for 30 min and subsequently 1 µL was injected. GC analysis was carried out using a ZB-35 column (Phenomenex, 30 m×250 µm ×0.25 µm; Aschaffenburg, Germany) with hydrogen as carrier gas at a constant pressure of 12 psi. Detection was done with a flame ionization detector (FID) and a hydrogen flow of 50 mL/min and an air flow of 450 mL/min. Extern standards of the relevant components were used for quantification. Coefficients of variation of independent experiments were below 5% (w/w). Each measurement was performed in triplicate. B: Structural formula of the investigated triterpenes occurring in birch bark extract (*Betula pendula*).
(TIF)

Figure S2 **TE (1 µg/mL) time dependently upregulates mRNA of the pro-inflammatory cytokines IL-1β (A) and TNF-α (B), TGF-β (C), the transcription factor Nrf2 (D), the antimicrobial peptide hBD3 (E) and the matrix-metalloproteinase MMP-2 (mRNA of MMP-9 was not upregulated) (F) in human primary keratinocytes.** Time course of mRNA expression measured by qRT-PCR. Values represent means of at least 2 independent experiments ± SEM. *p<0.05 and ***p<0.001 as indicated.
(TIF)

Figure S3 **TE and betulin have either none or a negligible effect on the production of the cytokines IL-1β, −4, −5, −7, −9, −10, −12, −13, −15, −17 and growth factors GM-CSF and VEGF in human keratinocytes after 24** **h incubation.** Cells were treated with TE (1 µg/mL) or betulin (0.87 µg/mL, which is in 1 µg/mL TE) for 24 h. Protein levels of the indicated mediators were determined in the supernatant by the Bio-Plex® Cytokine Assay. Values represent means of two independent experiments ± s.d.
(TIF)

Figure S4 **TE and betulin do not influence NFκB DNA binding in primary human keratinocytes.** Lane 1: Cells with TNF-α (4 ng/mL, for 30 min) as positive control, lane 2: unstimulated control, lanes 3–5: cells treated with TE (1 µg/mL) and lanes 6–8 treated with betulin (0.87 µg/mL, which is in 1 µg/mL TE) for the indicated times (2, 4, 6 h). Equal amounts of protein from total cell extracts were analyzed for NFκB-DNA binding activity in an electrophoretic mobility shift assay (EMSA). ○: NFκB-DNA complexes, •: non-specific binding to the probe, ◂: unbound oligonucleotide. The result of the EMSA was reproduced and one representative EMSA is shown.
(TIF)

Figure S5 **TE and betulin do not increase proliferation in primary human keratinocytes and in the WHM.** (A) BrdU-ELISA with primary human keratinocytes treated for 48 h with TE and the compounds at the respective concentrations. Values represent the means of at least 6 independent experiments ± SEM. (B) Proliferation in WHM 48 h after wounding and treatment with TE and the compounds. Values represent means of at least 7 independent experiments + SEM.
(TIF)

Figure S6 **Influence of TE (0.5 and 1 µg/mL), betulinic acid, oleanolic acid and erythrodiol (1, 10, and 100** **nM) on the actin cytoskeleton of primary human keratinocytes.** Cells were incubated on glass coverslips for 2 h with TE and the respective triterpenes for the indicated concentrations and the actin cytoskeleton was stained with phalloidin-rhodamine. 0.1% (V/V) DMSO was used as solvent control and CNF1 and CNFY as positive controls. Rows labelled with F show the impact on filopodia and lamellipodia and S the impact on stress fiber formation. A white arrow indicates the leading edge of the cell. Representative pictures of repeated experiments (n = 4) are shown.
(TIF)

Protocol S1 **The file contains all experimental data which is not shown in the manuscript.**
(DOC)
