# Supplementary figures and images for: From a Traditional Medicinal Plant to a Rational Drug: Understanding the Clinically Proven Wound Healing Efficacy of Birch Bark Extract
Source: PLoS One. 2014 Jan 22;9(1):e86147. doi: 10.1371/journal.pone.0086147 (PMC3899119; doi:10.1371/journal.pone.0086147)

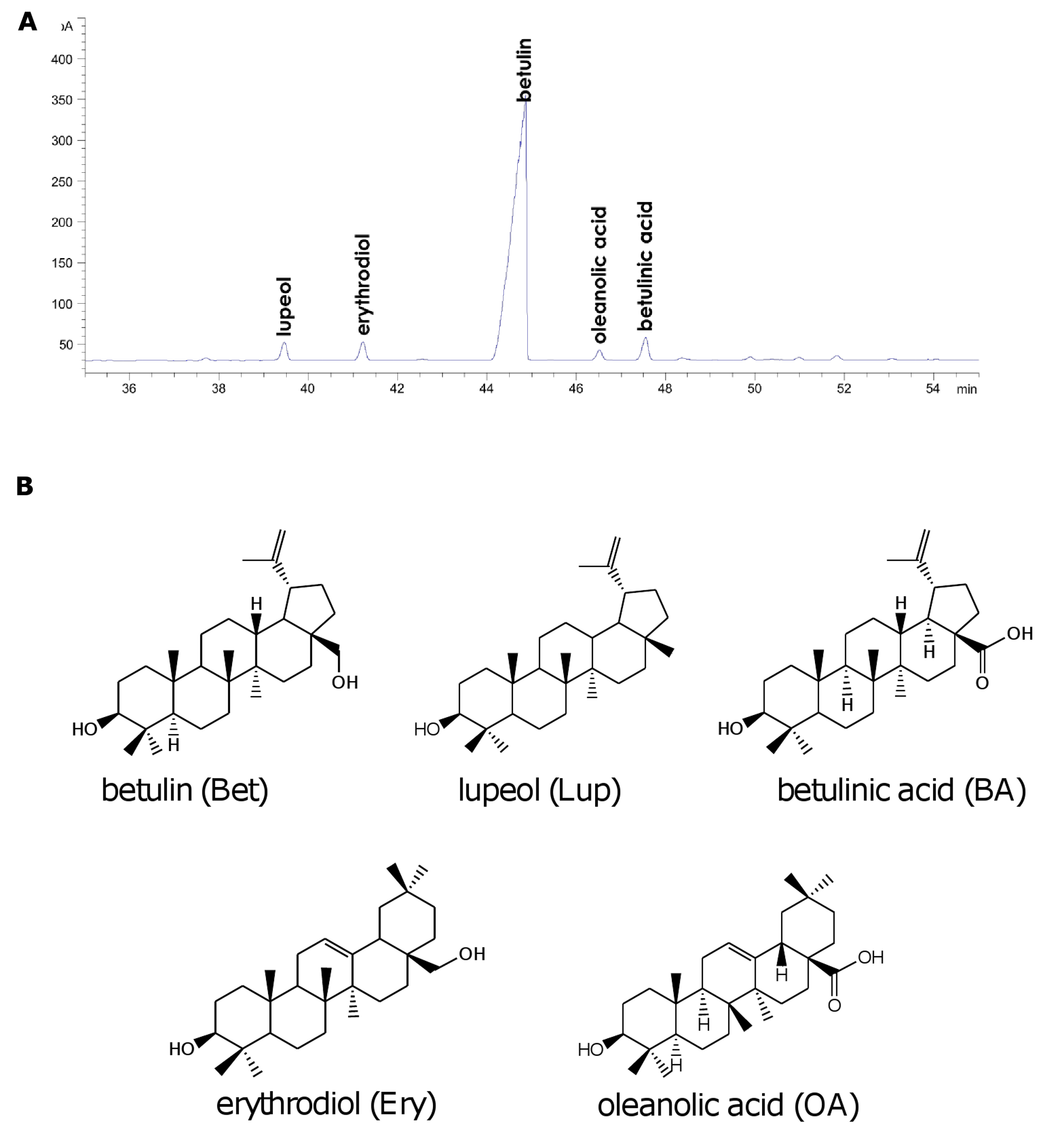

Supplement: Figure S1 — Quantitative analysis by gas chromatography and structures of triterpenes in birch bark. A: Quantitative composition of TE analyzed by gas chromatography [15]: betulin (86.85%), lupeol (3.94%), erythrodiol (0.77%), oleanolic acid (0.62%), betulinic acid (3.52%). In brief, 0.2 mg of TE was diluted in 500 µL tetrahydrofuran. This solution was silylated with 100 µL of silylating mixture (Silylating Mixture Fluka III; Buchs, Switzerland) at 80°C for 30 min and subsequently 1 µL was injected. GC analysis was carried out using a ZB-35 column (Phenomenex, 30 m×250 µm ×0.25 µm; Aschaffenburg, Germany) with hydrogen as carrier gas at a constant pressure of 12 psi. Detection was done with a flame ionization detector (FID) and a hydrogen flow of 50 mL/min and an air flow of 450 mL/min. Extern standards of the relevant components were used for quantification. Coefficients of variation of independent experiments were below 5% (w/w). Each measurement was performed in triplicate. B: Structural formula of the investigated triterpenes occurring in birch bark extract (Betula pendula). (TIF) [file pone.0086147.s001.tif]

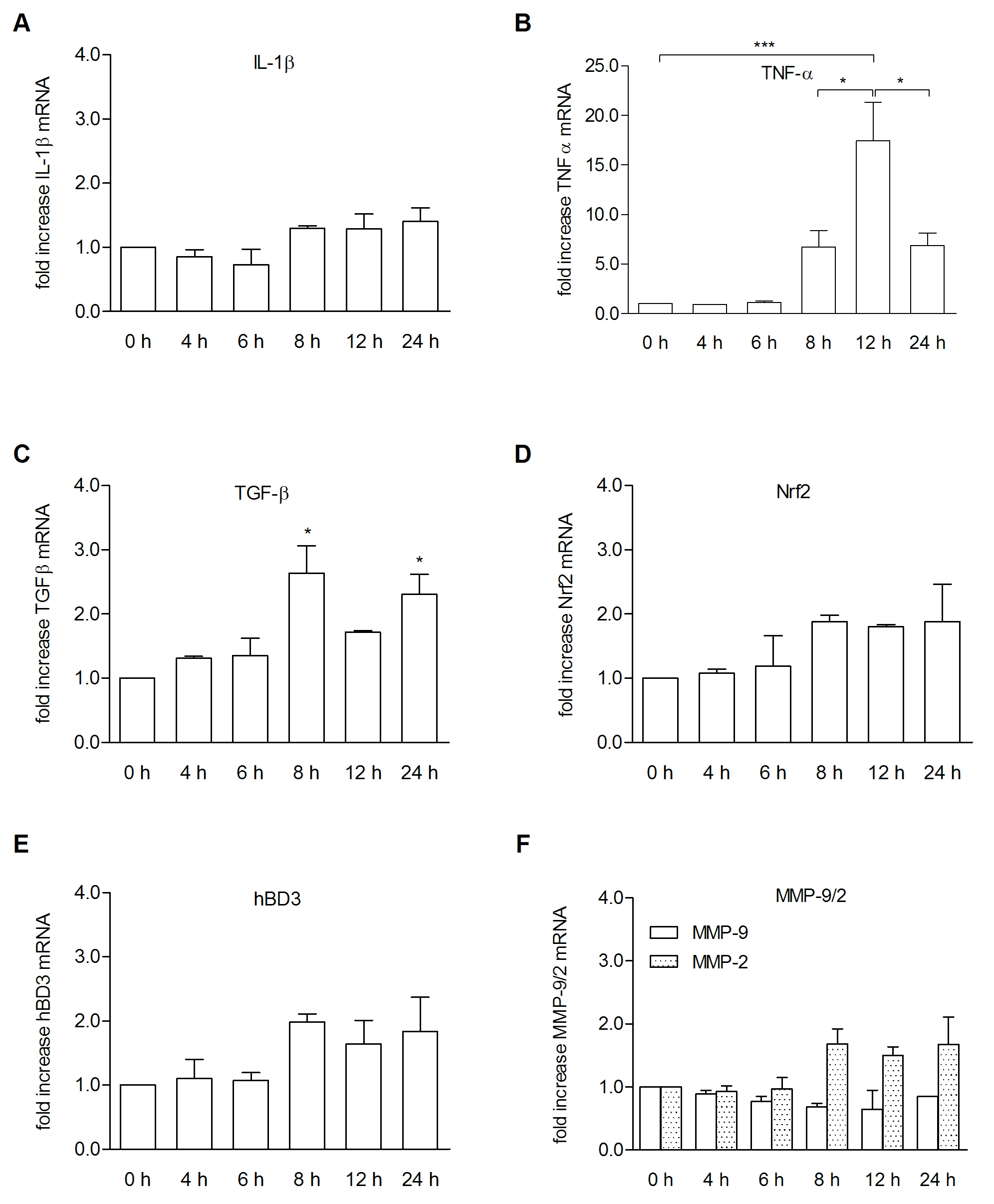

Supplement: Figure S2 — TE (1 µg/mL) time dependently upregulates mRNA of the pro-inflammatory cytokines IL-1β (A) and TNF-α (B), TGF-β (C), the transcription factor Nrf2 (D), the antimicrobial peptide hBD3 (E) and the matrix-metalloproteinase MMP-2 (mRNA of MMP-9 was not upregulated) (F) in human primary keratinocytes. Time course of mRNA expression measured by qRT-PCR. Values represent means of at least 2 independent experiments ± SEM. *p<0.05 and ***p<0.001 as indicated. (TIF) [file pone.0086147.s002.tif]

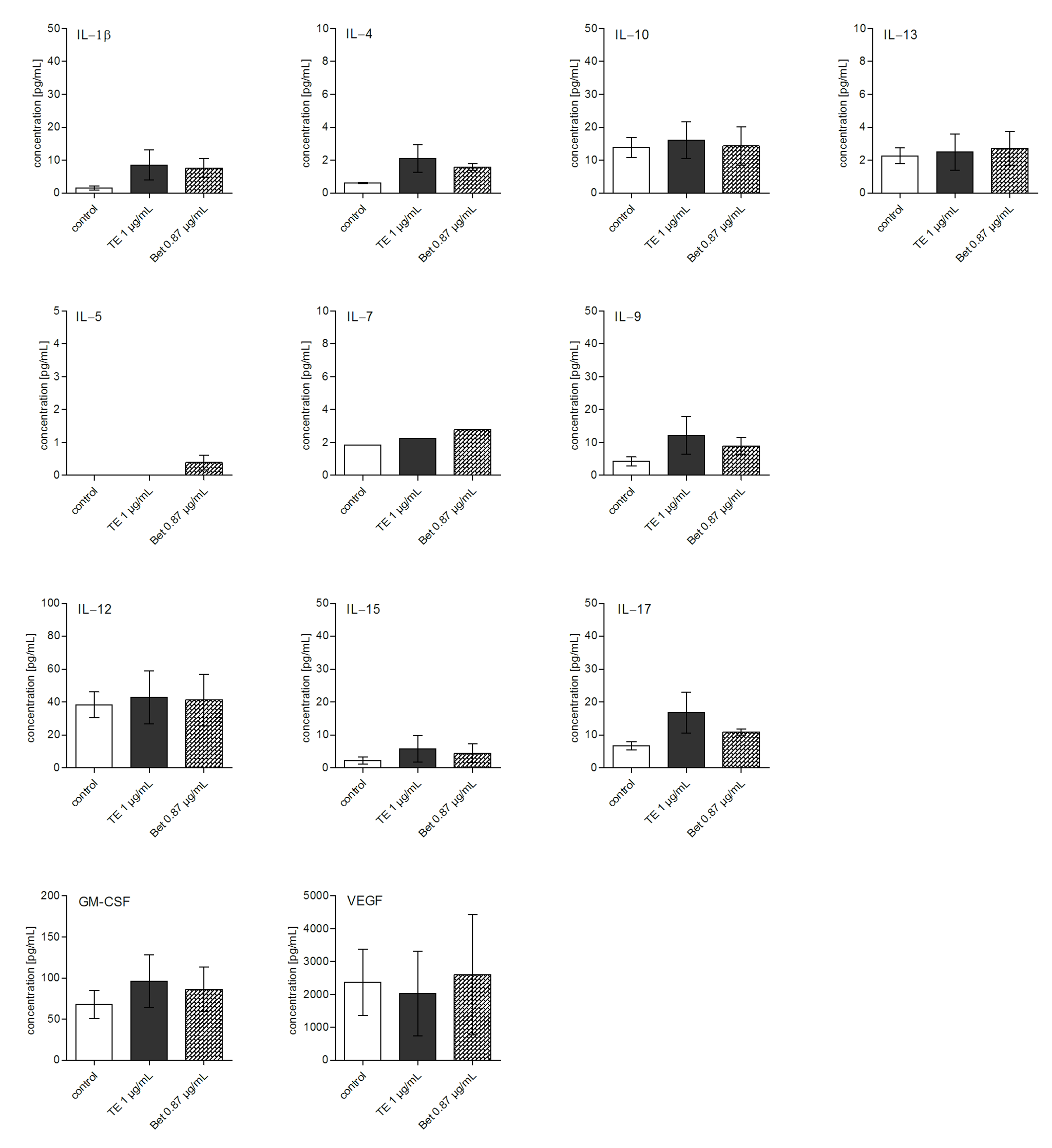

Supplement: Figure S3 — TE and betulin have either none or a negligible effect on the production of the cytokines IL-1β, −4, −5, −7, −9, −10, −12, −13, −15, −17 and growth factors GM-CSF and VEGF in human keratinocytes after 24 h incubation. Cells were treated with TE (1 µg/mL) or betulin (0.87 µg/mL, which is in 1 µg/mL TE) for 24 h. Protein levels of the indicated mediators were determined in the supernatant by the Bio-Plex® Cytokine Assay. Values represent means of two independent experiments ± s.d. (TIF) [file pone.0086147.s003.tif]

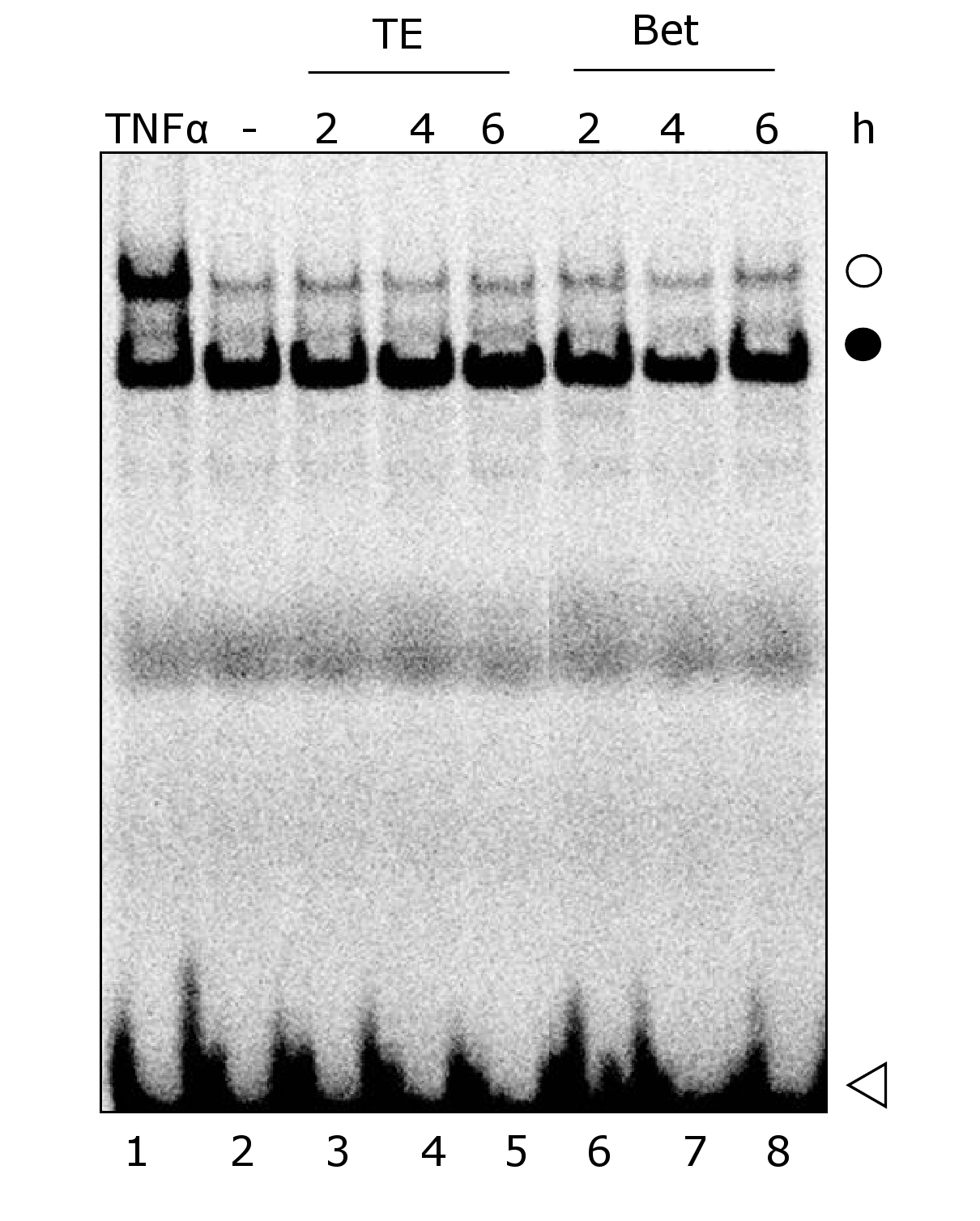

Supplement: Figure S4 — TE and betulin do not influence NFκB DNA binding in primary human keratinocytes. Lane 1: Cells with TNF-α (4 ng/mL, for 30 min) as positive control, lane 2: unstimulated control, lanes 3–5: cells treated with TE (1 µg/mL) and lanes 6–8 treated with betulin (0.87 µg/mL, which is in 1 µg/mL TE) for the indicated times (2, 4, 6 h). Equal amounts of protein from total cell extracts were analyzed for NFκB-DNA binding activity in an electrophoretic mobility shift assay (EMSA). ○: NFκB-DNA complexes, •: non-specific binding to the probe, ◂: unbound oligonucleotide. The result of the EMSA was reproduced and one representative EMSA is shown. (TIF) [file pone.0086147.s004.tif]

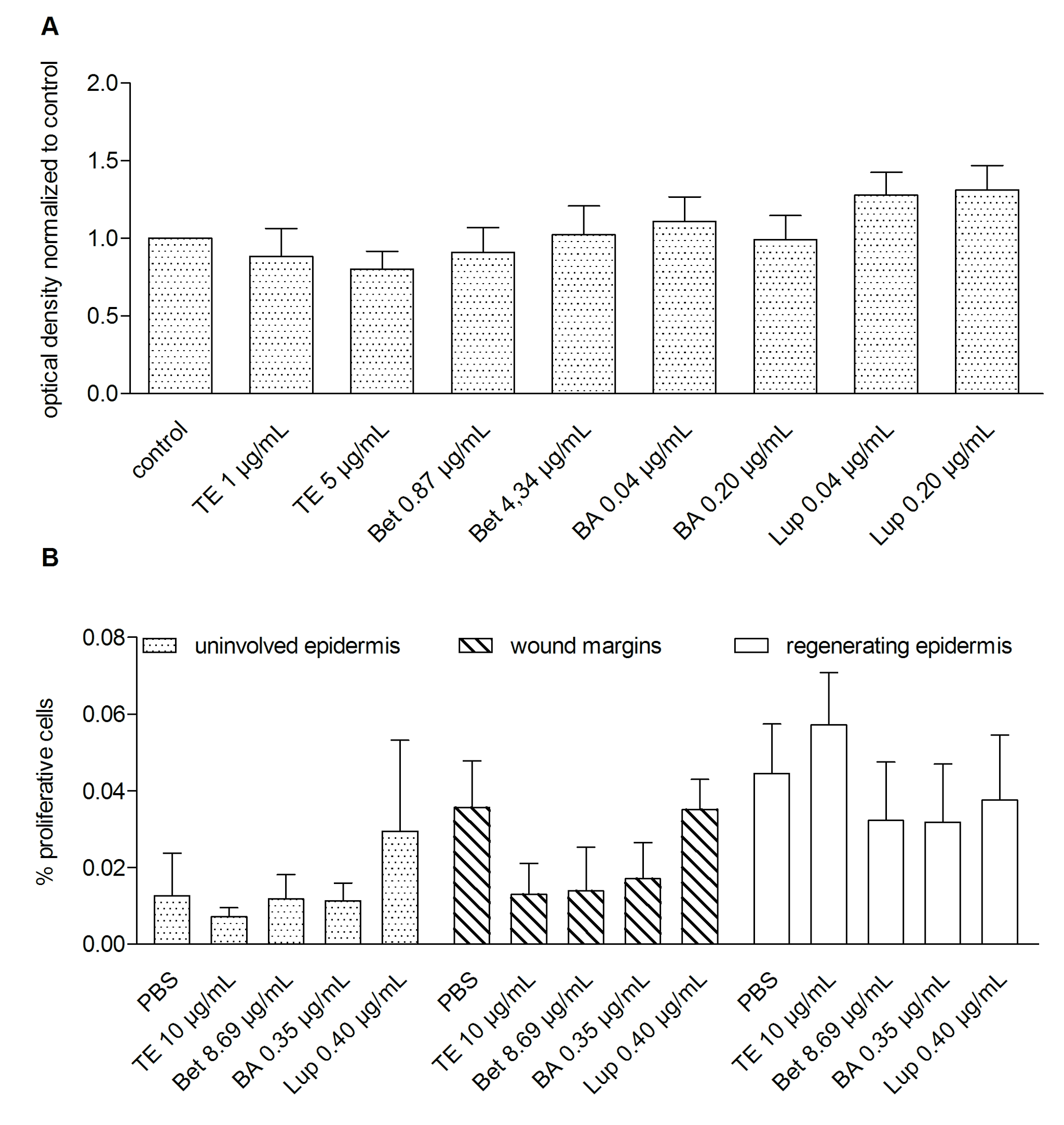

Supplement: Figure S5 — TE and betulin do not increase proliferation in primary human keratinocytes and in the WHM. (A) BrdU-ELISA with primary human keratinocytes treated for 48 h with TE and the compounds at the respective concentrations. Values represent the means of at least 6 independent experiments ± SEM. (B) Proliferation in WHM 48 h after wounding and treatment with TE and the compounds. Values represent means of at least 7 independent experiments + SEM. (TIF) [file pone.0086147.s005.tif]

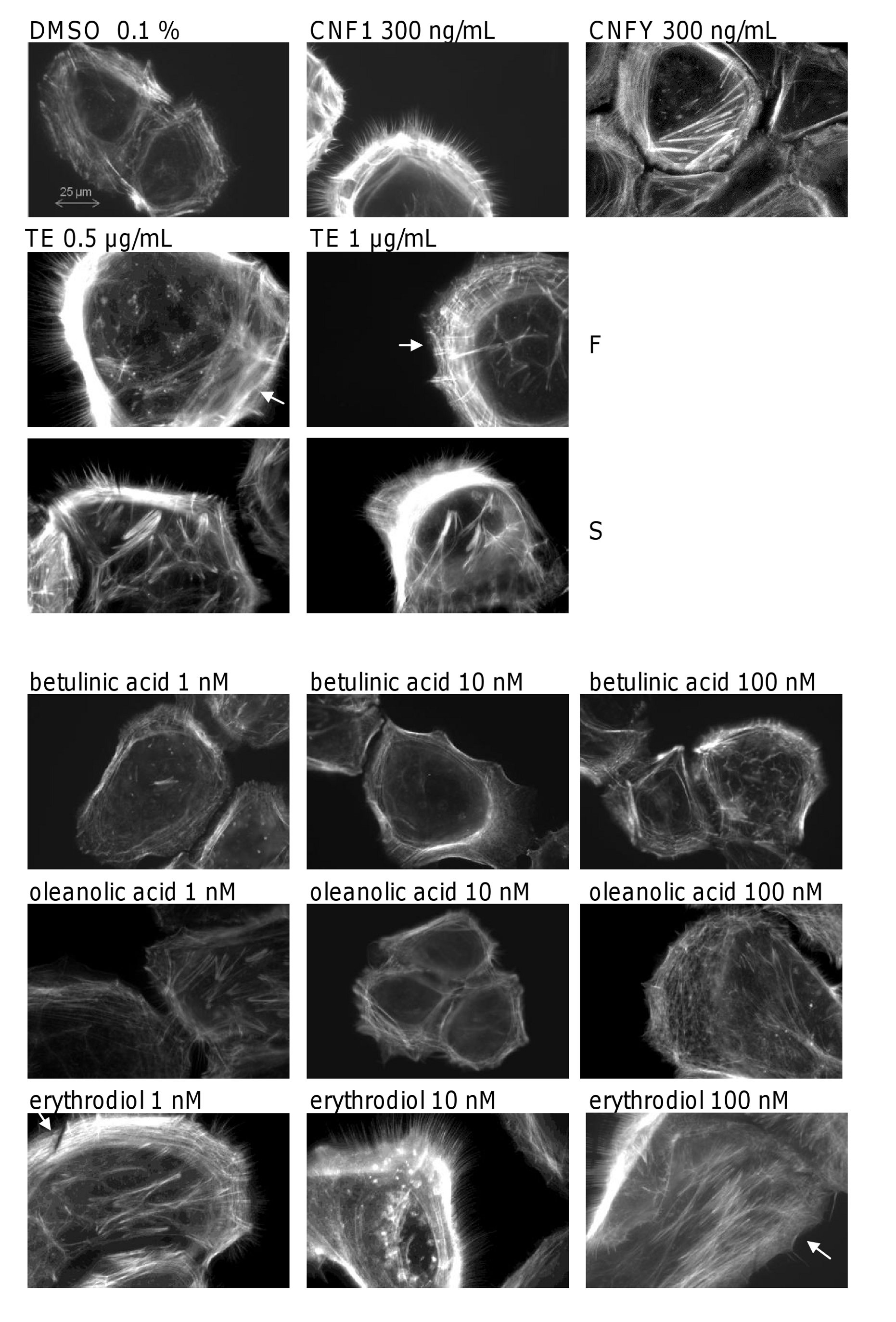

Supplement: Figure S6 — Influence of TE (0.5 and 1 µg/mL), betulinic acid, oleanolic acid and erythrodiol (1, 10, and 100 nM) on the actin cytoskeleton of primary human keratinocytes. Cells were incubated on glass coverslips for 2 h with TE and the respective triterpenes for the indicated concentrations and the actin cytoskeleton was stained with phalloidin-rhodamine. 0.1% (V/V) DMSO was used as solvent control and CNF1 and CNFY as positive controls. Rows labelled with F show the impact on filopodia and lamellipodia and S the impact on stress fiber formation. A white arrow indicates the leading edge of the cell. Representative pictures of repeated experiments (n = 4) are shown. (TIF) [file pone.0086147.s006.tif]
