# Supplementary material for: From a Traditional Medicinal Plant to a Rational Drug: Understanding the Clinically Proven Wound Healing Efficacy of Birch Bark Extract
Source: PLoS One. 2014 Jan 22;9(1):e86147. doi: 10.1371/journal.pone.0086147 (PMC3899119; doi:10.1371/journal.pone.0086147)
Supplement: Protocol S1 — The file contains all experimental data which is not shown in the manuscript. (DOC) [file pone.0086147.s007.doc]

**Supporting Information**

**From a Traditional Medicinal Plant to a Rational Drug: Understanding the Clinically Proven Wound Healing Efficacy of Birch Bark Extract.**

Sandra Ebeling^1^, Katrin Naumann^1^, Simone Pollok^2^, Tina Wardecki^1^, Sabine Vidal-y-Sy^2^, Juliana M. Nascimento^4,5^, Melanie Boerries^4,5,6^, Gudula Schmidt^3^, Johanna M. Brandner^2^, Irmgard Merfort^1^

^1^ Pharmaceutical Biology and Biotechnology, Albert-Ludwigs-University Freiburg, Freiburg, Germany

^2^ Department of Dermatology and Venerology, University Hospital Hamburg-Eppendorf, Hamburg, Germany

^3^ Institute for Experimental and Clinical Pharmacology and Toxicology, Albert-Ludwigs-University Freiburg, Freiburg, Germany

^4^ Institute of Molecular Medicine and Cell Research, Albert-Ludwigs-University Freiburg , Freiburg, Germany

^5^German Cancer Consortium (DKTK), Heidelberg, Germany

^6^German Cancer Research Center (DKFZ), Heidelberg, Germany

**Methods**

*Isolation and cultivation of primary normal human keratinocytes (NHK)*

Normal human skin keratinocytes (NHK) were derived from foreskin explants. Briefly, the skin was washed with PBS containing 8 % Antibiotic-Antimycotic® and 2 % Ciprobay® 200 mg solution for infusion and cut in small pieces. The separation of the epidermis was performed by 1 h incubation with trypsin/EDTA (0.05 %/0.02 %). NHKs were scraped with a scalpel and collected in PBS containing 2 % Ciprobay® solution and 10 % FCS and centrifuged at 4 °C. The cells were further cultivated in Keratinocyte SFM® supplemented with human recombinant EGF (rhEGF), bovine pituitary extract (BPE) and penicillin-streptomycin. These cells can be cultivated up to 5 passages. For cultivation, cells were washed in PBS and incubated with trypsin/EDTA (0.05 %/0.02 %) for 5 minutes. After centrifugation at 4 °C, cells were resuspended in Keratinocyte SFM® containing supplements and seeded into culture tissue flasks or wells at the desired concentrations. Cells were kept under a humidified environment with 5 % CO_2_ and 37 °C. Except for the BrdU- and cell scratch assay, the medium was always changed to a medium without supplements (Keratinocyte SFM® plus Penicillin-Streptomycin without rhEGF and BPE) 24 h prior to stimulation, in case of actin staining and Rho-GTPase pulldown experiments, cells were starved for 3 days. This treatment avoids activation of the cells by growth factors and hormones of the supplement.

*RNA isolation, cDNA synthesis and quantitative Real-Time PCR*

Total RNA from NHKs was isolated using the RNeasy® Plus Mini Kit from Qiagen according to the manufactures instructions. For each sample, 400.000 cells were used. The quantity and purity of RNA was determined by measuring the optical density at 260 and 280 nm. Subsequently, 1000 ng of total RNA was converted to single strand cDNA using the QuantiTect® Reverse Transcription Kit according to the manufactures instructions. The analysis of mRNA expression profiles was performed with qRT-PCR. In a 25 µl PCR reaction, 2 µl of cDNA (corresponding to 20 ng of total RNA input) was amplified in a Light Cycler® 480 using 2 x conc. LightCycler® 480 Probes Master, 50 nM primers and 100 nM probe for the 18S rRNA reference gene (fwd: 5’-CGGCTACCACATCCAAGG-3’, rev: 5’-CGGGTCGGGAGTGGGT-3’, probe: 5’-[HEX]-TTGCGCGCCTGCTGCCT-[TAM]-3’) or 300 nM primers and 200 nM probe for the HPRT1 reference gene (fwd: 5’-AGCACTGAACAGAAATAG-3’, rev: 5’-CCACCAATTACTTTTATATCG-3’, probe: 5’-[HEX]-TGGTCATTACAGTAGCTCTTC-AGTCT-[TAM]-3’) and 300 nM primers and 100 nM probe for the gene of interest. The following target gene primers and probes were used: human COX-2: fwd: 5’-GCCCTTCCTCCTGTGCC-3’, rev: 5’-AATCAGGAAGCTGCTTTTTACCTTT-3’, probe: 5’-[6-FAM]-ATGATTGCCCGACTCCCTTGGGTGT-[TAM]-3’; human IL-6: fwd: 5’-AGAAAACAACCTGAACCTT-3’ , rev: 5’-TGATGATTTTCACCAGGC-3’, probe: 5’-[6-FAM]-AGTCTCCTCATTGAATCCAGATTG-[TAM]-3’; human IL-8: fwd: 5’-CTCCATAAGGCACAAACT-3’, rev: 5’-AGTTCTTTAGCACTCCTT-3’, probe: 5’-[6-FAM]TCAGAGACAGCAGAGCACAC-[TAM]-3’; porcine COX-2: fwd: 5’-CAGCTCTACATTCAGAAG-3’ , rev: 5’-TAGTGGTCAAATCCTACA-3’, probe: 5’-[6-FAM]-CTCACCGCAACGCCTCTA-[TAM]-3’; porcine IL-6: fwd: 5’-GAACCCAGCTATGAACTC-3’ , rev: 5’-CCGAGGATGTACTTAATGA-3’, probe: 5’-[6-FAM]-TTCAGTCCAGTCGCCTTCTCC-[TAM]-3’; human IL-1β: fwd: 5’-GTACGATCACTGAACTGC-3’, rev: 5’-GTGGAGAGCTTTCAGTTC-3, probe: 5’-[6-FAM]-ATGGACCAGACATCACCAAGC-[TAM]-3’; human TNF-α: fwd: 5’-GTGAGGAGGACGAACATC-3’, rev: 5’-TGGTGGTCTTGTTGCTTA-3’, probe: 5’-[6-FAM]-AACCTTCCCAAACGCCTCCC-[TAM]-3’; human TGF-β: fwd: 5’-ACACCAACTATTGCTTCA-3’, rev: 5’-CTTGCGGAAGTCAATGTA-3’, probe: 5’-[6-FAM]-CGCACGCAGCAGTTCTTCTC-[TAM]-3’. human Nrf2: fwd: 5’-GTCACTTGTTCCTGATATTC-3’, rev: 5’-TCCCAAACTTGCTCAATG-3’, probe: 5’-[6-FAM]-AGTCTTCATTGCTACTAATCAGGCTCA-[TAM]-3’; human hDB3: fwd: 5’-CTAGCAGCTATGAGGATC-3’, rev: 5’-CTCTGCAATAATATTTCTGTAATG-3’, probe: 5’-[6-FAM]-CTTCTGTTTGCTTTGCTCTTCCTGT-[TAM]-3’. For relative quantification ΔC_T_ values of treated cells were referred to untreated control cells resulting in a ΔΔC_T_ value. The fold increase was calculated as 2^-ΔΔCT^.

*Determination of proliferation in WHM and cultured keratinocytes*

Proliferative (Ki67-positive) cells were detected by MIB-1 in WHM by immunofluorescence staining as described previously (Pollok et al., 2011). Total numbers of Ki67-positive were normalized to the total number of cells (visualized by DAPI staining) in the regenerating epidermis and at the wound margins, respectively. Wound margins were defined as the area of two rete ridges directly located at the wound. Both sides of the wounds were evaluated and the mean calculated. For the uninvolved epidermis two visual fields (0.25 mm^2^) were evaluated at both sides of the wound (n=7 different pigs). To determine proliferation in cultured keratinocytes, 8000 cells/well were seeded into 96-well plates, treated for 48 h with TE or the respective components and BrdU assays performed by using the BdrU-ELISA kit from Roche (Mannheim, Germany) according to the manufacturer’s instructions. Tests were performed in triplicates with 6-7 different primary keratinocyte cultures.

*Staining of the actin cytoskeleton*

To analyze the effect on the actin cytoskeleton, NHKs were stained with phalloidin-rhodamine and analyzed by fluorescence microscopy. Briefly, 10.000 NHKs per sample were seeded onto 10 mm glass coverslips in 12-well plates in Keratinocyte-SFM® and changed to starvation medium after 24 h for another 3 days to diminish basal stimulation of the actin cytoskeleton. After a stimulation of 2 h with the extract, the triterpenes and the positive controls CNFY (cytotoxic necrotizing factor of *Yersinia pseudotuberculosis*) and CNF1 (cytotoxic necrotizing factor 1 of *Escherichia coli*, 300 ng/ml each) cells were fixed with 400 µl 3.7 % formaldehyde and permeabilised with 0.1 % triton-X100 for 10 min followed by 6 times washing with PBS. The coverslips were then stained for 1 h with 1 µl phalloidin-rhodamine (0.2 Units) diluted in 24 µl PBS and washed 9 times with PBS. The coverslips were then placed on microscope slides and fixed with 3 µl Prolong Gold® Antifade reagent. Images were taken with an Axiovert fluorescence microscope (Zeiss).

*Preparation of the protein extracts for Western Blotting and EMSA*

Cell extracts were obtained by scraping NHK (600.000 in 10 cm^2^ petri dishes) in ice-cold PBS supplemented with 1% phosphatase inhibitor cocktail 2 and 4 % Complete® EDTA free protease inhibitor cocktail followed by a centrifugation step. In case of pSTAT3/STAT3 lysis was performed in extraction buffer containing 150 mM NaCl, 1 % sodium deoxycholate, 1 mM EDTA, 25 mM Tris pH 7.4, 1 % Triton X-100, 1 % phosphatase inhibitor cocktail 2 and 4 % Complete® EDTA free protease inhibitor cocktail. After incubation for 10 min on ice cells were centrifuged and the supernatant was collected, quickly-frozen in liquid nitrogen and stored at -80 °C. Cell extracts for p-p38/p38 Western blots and HuR were obtained by using totex lysis buffer containing 20 mM HEPES pH 7.9, 350 mM NaCl, 20 % glycerol, 1 % Nonidet NP-40, 1 mM MgCl_2_, 0,5 mM EDTA, 0,5 mM EGTA, 17 μg/mL Aprotinin, 1 mM DTT, 10 μg/mL Leupeptin, 10 μg/mL Pepstatin A, 1 mM PMSF, 5 mM NaF, 1 mM Na_3_VO_4_, 10 μl/mL PhosStop® (Roche, Mannheim, Germany). After incubation at 4 °C for 30 min under gently shaking, the supernatant was collected as described above and stored at -80 °C. Additionally to whole cell extracts cytosolic and nuclear extracts were prepared for HuR and NFκB as described by Schreiber et al. [2]

## *Electrophoretic mobility shift assay (EMSA)*

Nuclear protein extracts were prepared as described by Schreiber et al. [1]. For EMSAs, equal amounts of nuclear proteins (4 µg) were added to a reaction mixture containing 20 µg bovine serum albumin, 2 µg poly(dI-dC), 2 µl buffer D+ (20 mM HEPES, pH 7.9, 20 % glycerol, 100 mM KCl, 0.5 mM EDTA, 0.25 % Nonidet NP-40, 2 mM DTT, 0.1 % PMSF), 4 µl buffer F (20 % Ficoll 400, 100 mM HEPES, 300 mM KCl, 10 mM DTT, 0.1 % PMSF) and 25 ng of a [γ33P]-labeled oligonucleotide for NF‑κB made up to a final volume of 21 µl with distilled water. NF‑κB oligonucleotide (5’-AGT TGA GGG GAC TTT CCC AGG C-3’) was labeled using [γ33P]ATP (3000 Ci/mmol) and a T4 polynucleotide kinase. After 25 min of incubation at room temperature the samples were resolved through non-denaturing 6 % polyacrylamide gel electrophoresis and then the dried gel was exposed to an Imaging Plate (BAS-MS 2340, Fujifilm) overnight which was finally analyzed using a FLA-3000 (Fujifilm).

**References**

[1] Schreiber E, Matthias P, Muller MM and Schaffner W (1989) Rapid detection of octamer binding proteins with 'mini-extracts', prepared from a small number of cells. Nucleic Acids Res 17: 6419.

[2] Laszczyk M, Jager S, Simon-Haarhaus B, Scheffler A and Schempp CM (2006) Physical, chemical and pharmacological characterization of a new oleogel-forming triterpene extract from the outer bark of birch (betulae cortex). Planta Med 72: 1389-1395.
